# Deep brain stimulation rectifies the noisy cortex and irresponsive subthalamus to improve parkinsonian locomotor activities

**DOI:** 10.1038/s41531-022-00343-6

**Published:** 2022-06-20

**Authors:** Lan-Hsin Nancy Lee, Chen-Syuan Huang, Ren-Wei Wang, Hsing-Jung Lai, Chih-Ching Chung, Ya-Chin Yang, Chung-Chin Kuo

**Affiliations:** 1 Department of Physiology, National Taiwan University College of Medicine, Taipei, Taiwan; 2 Department of Neurology, Fu Jen Catholic University Hospital, New Taipei, Taiwan; 3 Department of Neurology, National Taiwan University Hospital, Taipei, Taiwan; 4 Graduate Institute of Biomedical Sciences, College of Medicine, Chang Gung University, Taoyuan, Taiwan; 5 National Taiwan University Hospital, Jin-Shan Branch, New Taipei, Taiwan; 6 Department of Biomedical Sciences, College of Medicine, Chang Gung University, Taoyuan, Taiwan; 7 Neuroscience Research Center, Chang Gung Memorial Hospital, Linkou Medical Center, Taoyuan, Taiwan; 8 Department of Psychiatry, Chang Gung Memorial Hospital, Linkou Medical Center, Taoyuan, Taiwan

**Keywords:** Basal ganglia, Parkinson's disease

## Abstract

The success of deep brain stimulation (DBS) therapy indicates that Parkinson’s disease is a brain rhythm disorder. However, the manifestations of the erroneous rhythms corrected by DBS remain to be established. We found that augmentation of α rhythms and α coherence between the motor cortex (MC) and the subthalamic nucleus (STN) is characteristically prokinetic and is decreased in parkinsonian rats. In multi-unit recordings, movement is normally associated with increased changes in spatiotemporal activities rather than overall spike rates in MC. In parkinsonian rats, MC shows higher spike rates at rest but less spatiotemporal activity changes upon movement, and STN burst discharges are more prevalent, longer lasting, and less responsive to MC inputs. DBS at STN rectifies the foregoing pathological MC-STN oscillations and consequently locomotor deficits, yet overstimulation may cause behavioral restlessness. These results indicate that delicate electrophysiological considerations at both cortical and subcortical levels should be exercised for optimal DBS therapy.

## Introduction

Parkinson’s disease (PD) is one of the most common movement disorders primarily due to degeneration of the dopaminergic neurons in the substantia nigra pars compacta (SNpc), and consequent deprivation of dopamine in the basal ganglia. The cardinal motor symptoms of PD include reduced and slowed motor activities (akinetic rigidity and bradykinesia). Deep brain stimulation of the subthalamic nucleus (STN-DBS) has been a choice of therapy for the motor symptoms in PD patients. The therapeutic effect of STN-DBS indicates that electrophysiological derangements may play an essential role in the symptomatic pathogenesis of PD. The increase of burst discharges in STN in single-unit recordings is a well-recognized pathophysiologic marker in PD and in animal models of dopamine deprivation^1–3^. Our previous studies have shown that the increase of burst discharges of STN has a direct causal relation with the locomotor deficits of parkinsonian rats^4–8^. At the cellular level, injection of local currents mimicking DBS at STN depolarizes STN neurons^4^. Neuronal depolarization inactivates T-type Ca^2+^ channels and/or Na^+^ channels and thus suppresses burst discharges to ameliorate locomotor deficits^4–6^. At the circuit level, STN receives major excitatory afferent inputs directly from the motor cortex (MC). Anatomically and functionally, deranged cortico-subthalamic transmission was reported in animals and patients of PD^9–15^. Also, high-frequency stimulation of the cortico-subthalamic hyperdirect pathway could result in parkinsonian locomotor deficits even in normal animals^16^. We have recently demonstrated that MC is directly involved in the genesis of excessive STN burst discharges^17^. Moreover, excessive STN burst discharges could in turn trigger highly correlative MC activities and apparent tremulous behaviors in dopamine-depleted animals^17,18^. We therefore postulate that the MC-STN axis, a pathway being relatively neglected before^19–21^, may play a pivotal role of feedforward modulation in the symptomatic pathogenesis of PD.

In addition to the excessive burst discharges in STN, increase in β power and β coherence (“β augmentation”) in local field potentials (LFP) in the cortico-basal ganglia-thalamic circuits was considered as another electrophysiological hallmark in PD^22–25^. β augmentation may signal a large-scale or network abnormality in PD and seems to play a less certain role in parkinsonian locomotor deficits if compared to the increase of STN burst discharges. For instance, although several studies suggested a role of β augmentation in the deranged motor control in PD^23,25–31^, other studies found more complexities in correlating β oscillations with different motor deficits in PD^7,32–35^. Although a dampened event-related desynchronization (ERD) of β oscillations upon movement was reported in PD^29,36,37^, there were inconsistent findings among different movements or in different situations^38–40^. Also, phasic increase of β oscillation was induced by instruction cues, but not by movement initiation or immobilization^41^. Moreover, STN-DBS that ameliorates parkinsonian locomotor deficits may or may not suppress MC-STN β augmentation^42–46^. Our previous findings also showed that parkinsonian locomotor activities are directly correlated with STN burst discharges rather than β augmentation^7^. Except for the β band, the changes in LFP power in the other (e.g. θ-α or γ) spectra and their pathophysiological roles in PD have received less attention and similar controversies exist^22,33,37,47–49^. For example, there could be suppression or enhancement of α or θ-α oscillations after successful STN-DBS treatment^42–44,50^. The documented electroencephalograms (EEG) or LFP manifestations in response to STN-DBS have been descriptive, with inconsistent reports on the changes in cortical evoked potentials, and in α, β, or the other band powers^51–57^. We postulate that the controversies may partly arise from the insufficiently differentiated behavioral states in those studies (e.g. the whole period of “movement” may include short intervals of “rest”). The lack of multi-unit (MU) or single-unit (SU) findings underlying the descriptive LFP changes may also preclude a mechanistic link of LFP power spectrum changes to parkinsonian locomotor deficits and thus a comprehensive functional view of the foregoing proposal of the pivotal role of the MC-STN axis in the symptomatic pathogenesis of PD. With meticulous differentiation of the intervals of movement from rest and concomitant recordings of both LFP and MU electrophysiological activities in a parkinsonian animal model^58–60^, we found a decrease in LFP α power but relatively preserved “prokinetic α augmentation” in parkinsonian animals. Correlatively, the MU activities in MC are markedly increased at rest, but fail to show more frequent changes upon movement, which may be partly ascribed to the more prevalent, longer, but less variable STN burst discharges in response to cortico-subthalamic drives upon dopaminergic deprivation.

## Results

### There is a general decrease in α power in MC and STN in parkinsonian rats

**Fig. 1:**
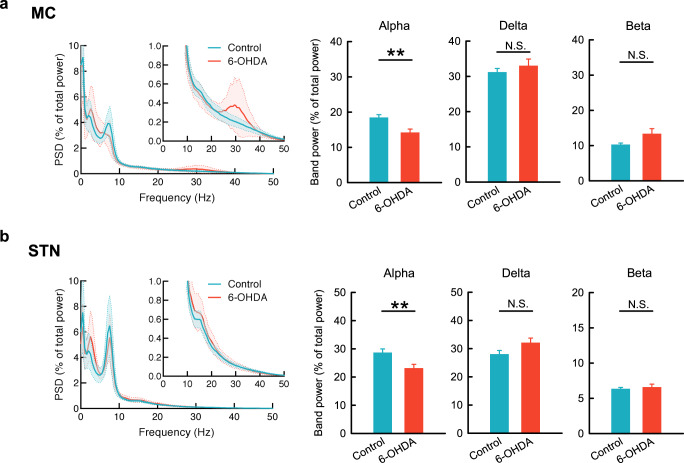
The basic rhythms of MC and STN in control and parkinsonian rats. The average PSD spectra of 29 and 20 continuous 5-min LFP recordings from 10 control (blue) and 11 parkinsonian (6-OHDA, red) rats, respectively. The open field test (OFT) reveals a significantly higher peak in the α band (7–10 Hz) in control than in parkinsonian rats in both MC (**a**) and STN (**b**). Meanwhile, there is a lower δ band (1–4 Hz) power in both MC and STN in control than in parkinsonian rats, although the differences are statistically insignificant. In contrast, a peak oscillation in β band (20–40 Hz) in parkinsonian but not in control rats is noted only in MC. PSD spectra were presented as mean (solid curves) ± standard deviation (shaded areas). Data in the bar plots were presented as mean ± standard error of mean (S.E.M.). Data were analyzed with Mann–Whitney *U* tests. **p < 0.01, N.S., nonsignificant.

The general tendency of LFP oscillations in MC and STN was characterized in control and 6-OHDA hemiparkinsonian free-moving rats. In control rats, the average power spectrum density (PSD) reveals a prominent peak oscillation in the α (7–10 Hz) range both in MC and in STN (Fig. 1a, b). In 6-OHDA hemiparkinsonian rats, the α peak almost disappears in MC and significantly decreases in STN compared to control. Parkinsonian rats also tend to show a β (20–40 Hz) peak in MC but not STN, and a higher δ (1–4 Hz) peak in STN and also MC, although the differences in band powers from control are not statistically significant. These results indicate that the most characteristic difference in LFP oscillations in the MC-STN axis between control and parkinsonian rats is in the α, but not β or δ, frequency bands (also see Supplementary Fig. 1).

### The α band power in MC and STN increases upon movement

**Fig. 2:**
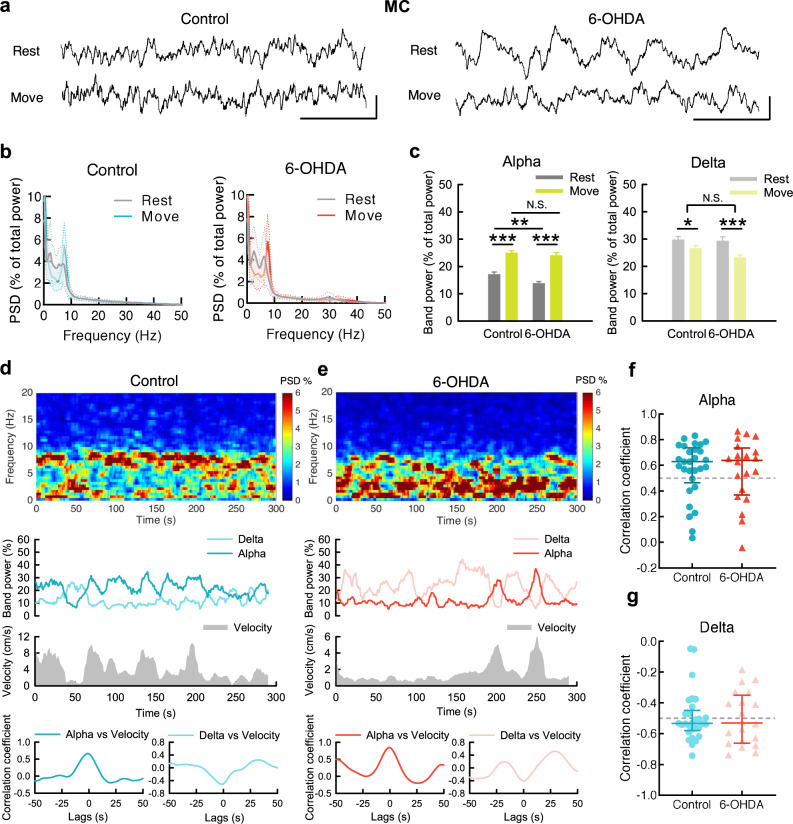
The changes of MC rhythms upon movement in control and parkinsonian rats. **a** Sample MC LFP recordings during rest and movement from a control rat (left) and a parkinsonian rat (6-OHDA, right). Scale bar represents 500 ms/100μV. **b** Average PSD spectra in MC during 10-s continuous rest or movement show a marked increase of α power and decrease of δ power from rest to movement in both control and parkinsonian rats (*n* = 52 and 50 matched segments of rest and movement from 10 control and 11 parkinsonian rats, respectively). **c** Band power analysis from part **b**. **d**, **e** Power spectrograms (top) and the temporal changes of MC α and δ band powers (middle, upper) as well as concomitant moving velocities (middle, lower) from a sample 5-min recording in OFT in control (**d**) and in parkinsonian rats (**e**). Cross-covariance analysis between time-varying band power and moving velocity reveals an evident peak of correlation coefficient at zero time lag in α band (bottom, left), but a valley in δ band (bottom, right) in control and parkinsonian rats. Each series of data contain one data point per second (300 data points in total). **f** Cross-covariance analysis from all 5-min recordings shows that most of control and parkinsonian rats have a strong correlation coefficient (>0.5) between MC α power and moving velocity at zero time lags. Correlation coefficient >0.5: control = 21/29 (72%), parkinsonian = 14/20 (70%); median of correlation coefficient, control: 0.63, parkinsonian: 0.64. **g** Similar analysis to that in (**f**) shows no positive but negative correlation between δ power and moving velocity. Correlation coefficient < −0.5: control = 19/29 (66%), parkinsonian = 11/20 (55%); median of correlation coefficient, control: −0.53, parkinsonian: −0.53). PSD spectra were presented as mean (solid curves) ± standard deviation (shaded areas). Data in bar plot were presented as mean ± S.E.M. Data in (**c**) were analyzed with 2 × 2 mixed model ANOVA and simple main effect tests for pairwise comparison. Each horizontal line reports the simple main effect, and the square bracket reports the main effect of control vs. 6-OHDA in ANOVA. *p < 0.05, **p < 0.01, ***p < 0.001, N.S., nonsignificant.

**Fig. 3:**
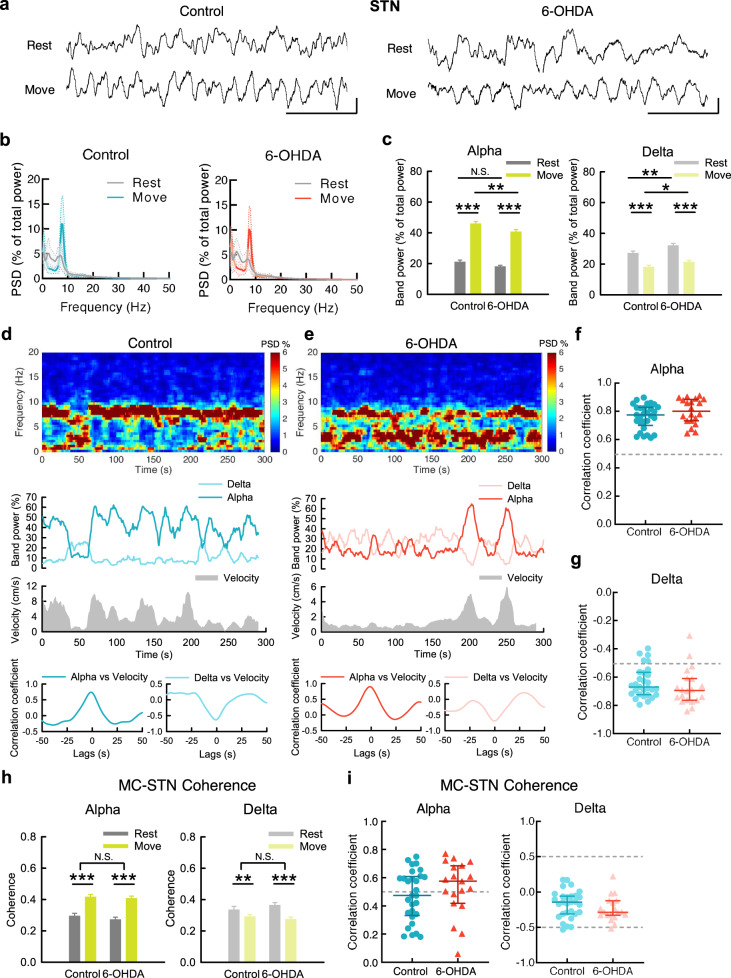
The changes of STN rhythms upon movement in control and parkinsonian rats. **a**–**g** Similar experiments and analyses to those in Fig. 2 were done on STN. 52 and 50 matched segments of rest and movement were analyzed for control and parkinsonian rats, respectively. Band power analysis reveals significant increase of α power and decrease of δ power from rest to movement in both control and parkinsonian rats (**c**). Correlation coefficient > 0.5: control = 29/29 (100%), parkinsonian = 20/20 (100%); median of correlation coefficient, control: 0.78, parkinsonian: 0.8, between STN α power and moving velocity at zero time lags (**f**). Correlation coefficient < −0.5: control = 25/29 (86%), parkinsonian = 18/20 (90%); median of correlation coefficient, control: −0.66, parkinsonian: −0.68, between STN δ power and moving velocity (**g**). Scale bar represents 500 ms/100μV. **h** Average MC-STN coherence in α band reveals a significant increase from rest to movement in both control and parkinsonian rats, accompanied by a decrease of δ coherence in parkinsonian rats. Either α or δ coherence shows no difference between control and 6-OHDA rats. **i** Most control and parkinsonian rats show a positive correlation, or even a strong positive correlation, coefficient between α coherence and moving velocity in cross-covariance analysis (correlation coefficient >0.5: control: 14/29, parkinsonian: 14/20, median of correlation coefficient, control: 0.47, parkinsonian: 0.55). In contrast, almost all of the control and parkinsonian rats show no strong correlation between MC-STN δ coherence and moving velocity (correlation coefficient < −0.5: control: 1/29, parkinsonian: 1/20; median of correlation coefficient, control: −0.14, parkinsonian: −0.28). PSD spectra were presented as mean (solid curves) ± standard deviation (shaded areas). Data in bar plot were presented as mean ± S.E.M. Data in (**c**) and (**h**) were analyzed with 2 × 2 mixed model ANOVA and simple main effect tests for pairwise comparison. Each horizontal line reports the simple main effect, and each square bracket reports the main effect of control vs. 6-OHDA in ANOVA. *p < 0.05, **p < 0.01, ***p < 0.001, N.S., nonsignificant.

**Fig. 4:**
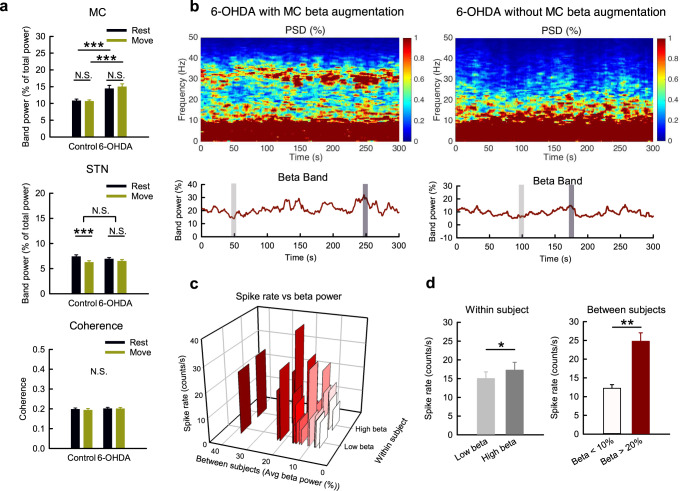
The association of MC β augmentation with higher spike activities but not locomotor activities in parkinsonian rats. **a** β band power analysis from the 10-s segments in Figs. 2, 3 (*n* = 52 and 50 matched segments of rest and movement from 10 control and 11 parkinsonian rats, respectively). **b**–**d** Power spectrograms (tops) and the temporal changes of MC β band powers (bottoms) from sample recordings demonstrate the typical presentation of parkinsonian rats with MC β augmentation or not (**b**, left and right, respectively). Among the 20 continuous 5-min recordings from 11 parkinsonian rats, a peak in β range frequency was noted in 9 recordings (45%) but not in the other 11 recordings (55%). Each recording was further divided into two 10-s segments, with highest (dark gray bar) or lowest (light gray bar) β band power, as the high β group (“High beta”) and low β group (“Low beta”), respectively, for multi-unit analysis. The 3D-bar plot reveals average spike rates from 7 leads grouping according to the level of β power in each recordings of parkinsonian rats, and shows that higher β power is associated with a higher spike rate in both within-subject comparison and between-subject comparison (**c**). There is a significant higher MC MU spike rate in “High beta” group than in “Low beta" group (*n* = 17 from 8 parkinsonian rats) (**d**, left). There is also a higher spike rate in recordings with extreme β augmentation (average β power > 20%) than in recordings with scarcity of β (average β power < 10%), *n* = 4 from 2 rats in both groups (each group had *n* = 8 segments from “High beta” and “Low beta” groups) (**d**, right). Data were presented as mean ± S.E.M. Data in (**a**) were analyzed with 2 × 2 mixed model ANOVA and simple main effect tests for pairwise comparison. Each horizontal line reports the simple main effect, and each square bracket reports the main effect of control vs. 6-OHDA in ANOVA. The data in the right and left panels in (**d**) were analyzed with Mann–Whitney *U* tests and Wilcoxon signed-rank tests, respectively. *p < 0.05, **p < 0.01, ***p < 0.001, N.S., nonsignificant.

Figure 1 illustrates the general changes in LFP oscillations irrespective of the behavioral correlates. We then characterized the behavior-specific changes in LFP to explore the functional connotation of the segregated bands of LFP oscillations (Fig. 2). We found that movement is associated with a sharp increase in α power in MC in both control and parkinsonian animals (Fig. 2a–c). In addition, there is a strong positive temporal correlation between MC α power and moving velocity in both animals (Fig. 2d–g). It is noteworthy that α power is lower in parkinsonian than in control animals especially at rest (but not so much during movement, Fig. 2c). Corresponding to the increase in α power, there is a decrease in δ power in MC and a negative temporal correlation between locomotor activities and the δ power during movement (Fig. 2a–g). The close correlation between MC α power and locomotor activities is well in line with the general decrease of such an oscillatory pattern in parkinsonian rats (e.g. Fig. 1). There is also a dramatic increase in α power and decrease in δ power in STN upon movement in both control and parkinsonian rats (Fig. 3). On the other hand, β power is not manifestly associated with changes in locomotor activities in both control and parkinsonian animals (Fig. 4). The prominent phasic increase in MC-STN α coherence (Fig. 3h, i) not only lends a strong support for the “α augmentation” upon movement, but also implicates a close mechanistic correlation or interaction between MC and STN for the generation of the prokinetic α rhythms. If compared with the cases in MC (Fig. 2f, g), the positive correlation of movement to the LFP α power is even more prominent in STN (Fig. 3f, g). The STN discharge pattern thus seems to be an even better marker signaling the state of locomotor execution than MC. However, as the final motor commands are directly from MC, STN activities shall be closely coherent to MC activities. In this regard, the highly coherent prokinetic α rhythms in the MC-STN axis are especially noteworthy (Supplementary Fig. 2c, d). The erroneous STN discharges, including the excessive bursts, may then contribute to an erroneous discharge pattern of MC (and vice versa via the cortico-subthalamic fibers or hyperdirect pathway to complete the “vicious cycle”^17^) to play a critical role in the pathophysiological motor control in PD.

### Dopamine deprivation leads to dissociation of MC from STN in multi-unit activities

**Fig. 5:**
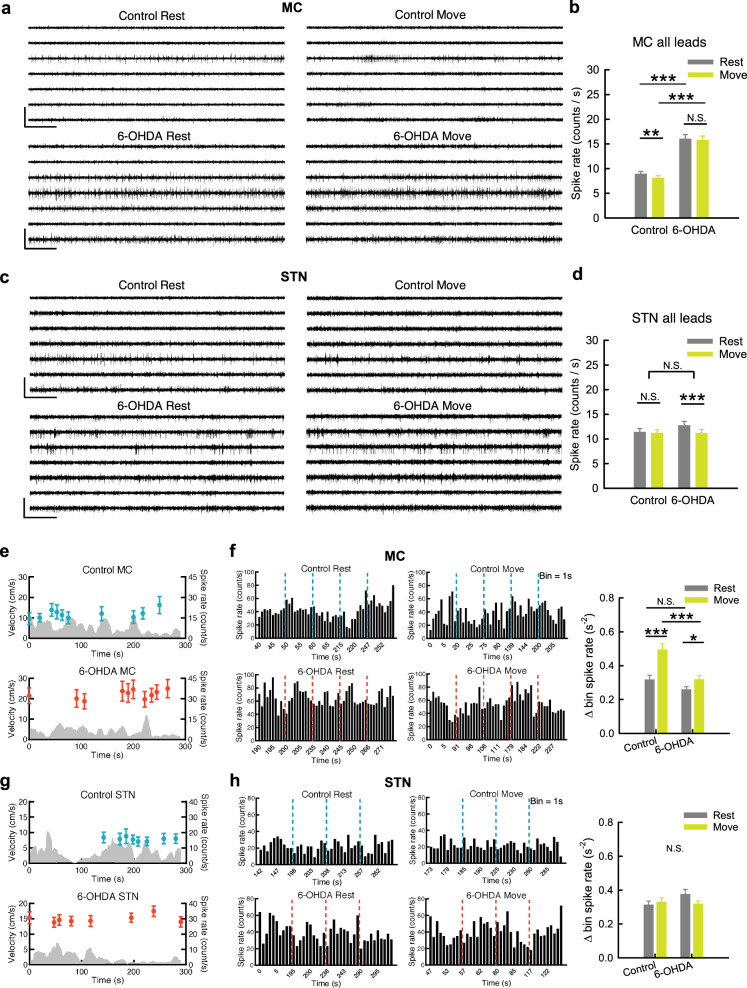
Higher overall rates and less time-dependent changes of MC multi-unit activities associated with movement in parkinsonian rats. **a** Sample sweeps of multi-unit (MU) recordings from 7-lead arrays in MC from a control (top) and a parkinsonian rat (6-OHDA, bottom). **b** The average MU spike rates in MC are significantly higher in parkinsonian than control rats. *n* = 364 and 350 (52 and 50 segments × 7 leads) rest and movement pairs from 10 control and 11 parkinsonian rats, respectively. **c**, **d** Similar experiments and analyses to (**a**) and (**b**) were done on STN. *n* = 364 and 350 pairs in control and parkinsonian rats, respectively. **e** The temporal profiles of the average MC MU spike rates in a single OFT trial in one control (blue dots) and one parkinsonian rat (red dots) from the same leads in (**a**) reveal no correlation with the moving speed (gray areas) in both cases (each point of the spike rate is plotted at the center of time of a segment in the OFT trial, and represents the average spike rate of the 7 leads in a segment). **f** Rate histograms from one sample lead from all segments in (**e**) reveal apparently more changes in MC spike rates between adjacent bins from rest to move in control (left panel, upper) than in parkinsonian rats (left panel, lower). The average bin spike rate change (bin = 1 s) in all sampled segments. *n* = 35 (5 segments × 7 leads) rest and movement pairs from one control rat and one parkinsonian rat, respectively. **g**, **h** Similar experiments and analyses to (**e**) and (**f**) were done on STN. *n* = 24 and 28 pairs in control and parkinsonian rat, respectively. ^#^One lead in the sample control STN segments was excluded from statistics due to the extraordinarily low spike rate (<1 Hz). Scale bars represent 500 ms/500μV. Data were presented as mean ± S.E.M. Data were analyzed with 2 × 2 mixed model ANOVA and simple main effect tests for pairwise comparison. Each horizontal line reports the simple main effect, and each square bracket reports the main effect of control vs. 6-OHDA in ANOVA. *p < 0.05, **p < 0.01, ***p < 0.001, N.S., nonsignificant.

LFP are time-dependent potential changes due to changes in momentarily summed local currents and thus cellular activities. To explore the mechanistic basis underlying the decrease in MC LFP α power in parkinsonian animals, we examined the multi-unit (MU) activities in MC and STN. Surprisingly, the overall MU activities (spike rates) in MC, but not those in STN, increase ~2 fold in parkinsonian than in control animals during either rest or movement (Fig. 5 a–d). Moreover, the MU activities in MC do not increase upon movement than at rest in either control or parkinsonian animals (there is even a slight decrease of spike rates from rest to movement in control rats, Fig. 5a, b). The marked increase in MU activities in MC but not in STN indicates an interesting electrophysiological dissociation between MC and STN on top of the preserved prokinetic MC-STN α coherence in PD (also refer to Figs. 2, 3), a finding probably relevant to the less responsive STN to the direct drive from the more active MC. The more active, or “more noisy”, MC characterized by an increase in MU activities could result in more frequent time-dependent changes in LFP and thus β augmentation in MC than STN, in parkinsonian animals (Figs. 1, 4a). Consistently, β augmentation tends to happen with higher MU activities within the same and between different subjects (Fig. 4b, c), although the correlation between β power and overall MU activities is not tight. This suggests additional factors contributory to β augmentation, which is therefore a relatively inconsistent electrophysiological feature in PD (Fig. 4, see also “Discussion”).

### Movement-associated dynamic changes in spatiotemporal patterns of MC discharges are impaired in parkinsonian rats

**Fig. 6:**
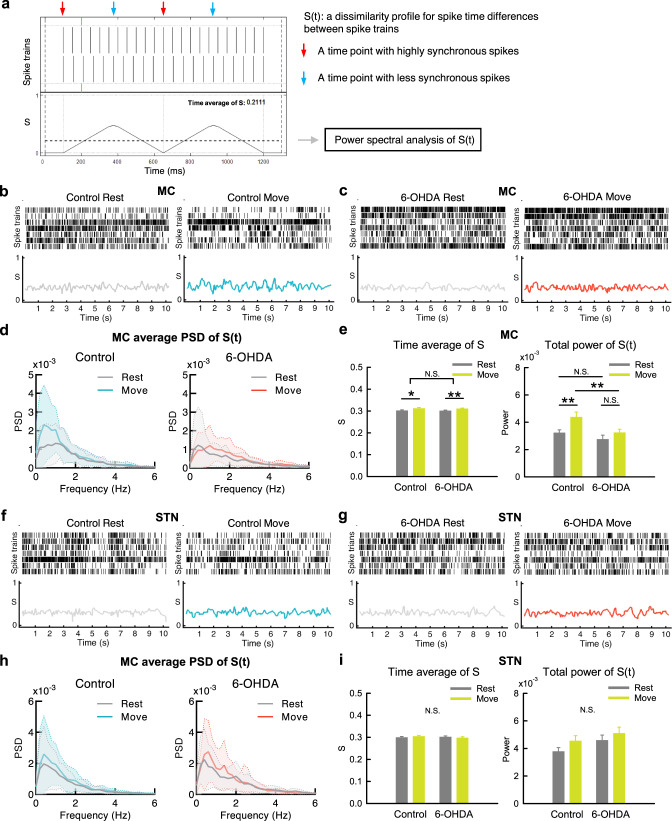
Less spatiotemporal changes in MC discharges from rest to movement in parkinsonian rats. **a** A diagram for the SPIKE-distance, a time-dependent dissimilarity score (S), in 2 simulate spike trains. The waveform denotes temporal profile of S (S(t)), and the dotted line denotes the time average of S. S(t) for 7 spike trains is calculated by averaging all pairwise S(t) (see “Method”, and also refer to Kreuz et al.^112^). **b**, **c** Sample raster plots of multi-unit spikes in MC and concomitant dissimilarity score (S), from a segment at rest (left) and the other one during movement (right) in a control rat (**b**) and a parkinsonian rat (6-OHDA, **c**). Note the apparently larger temporal changes in S during movement than at rest in control but not in parkinsonian rats. **d** The average power-frequency spectra of the whole temporal profile of S (S(t)) in all segments. *n* = 27 at rest and *n* = 23 during movement in control, and *n* = 35 at rest and *n* = 37 during movement in parkinsonian rats, from 10 control and 11 parkinsonian rats, respectively. Note the markedly enhanced peak at ~0.5 Hz from rest to movement in control but not in parkinsonian rats. **e** The time average of the dissimilarity score (S) of the 10-s segments in MC (left panel), and the total powers (0–250 Hz) of the spectral analyses from segments in part **d** (right panel). **f**–**i** Similar analyses to (**b**–**e**) were done on STN. *n* = 33 at rest and *n* = 34 during movement in control, and *n* = 22 at rest and *n* = 21 during movement in parkinsonian rats, from 10 normal and 11 parkinsonian rats, respectively (**h**, **i**). PSD spectra were presented as mean (solid curves) ± standard deviation (shaded areas). Data in the bar plot were presented as mean ± S.E.M. Data in (**e**) and (**i**) were analyzed with 2 × 2 independent model ANOVA and simple main effect tests for pairwise comparison. Each horizontal line reports the simple main effect, and each square bracket reports the main effect of control vs. 6-OHDA in ANOVA. *p < 0.05, **p < 0.01, N.S., nonsignificant.

Consistent with the essential lack of changes in overall MU discharges in MC from rest to movement (Fig. 5a–d), detailed behavioral analysis shows no correlation between the moving velocity and the overall MC spike rate (Fig. 5e). However, movement is clearly accompanied by an increase in temporal (time-dependent) changes of MU spike rates in MC, a feature more manifest in control than in parkinsonian rats (Fig. 5f). In STN, neither overall MU spike rates nor their dynamic temporal changes are associated with movement (Fig. 5g, h). In addition to temporal profiles, we also investigated the changes in spatial profiles of neural activities associated with movement (Fig. 6). Dissimilarity analysis of the spike trains among 7 different recording spots in MC reveals more frequent spatial pattern changes in MU activities from rest to movement in control than in parkinsonian rats (Fig. 6a–e). This is as if the dynamic changes in regional activity patterns for the formation of appropriate cortical motor programs are reduced in parkinsonian rats. The markedly increased overall MC activities in parkinsonian rats (Fig. 2) therefore could signal significant contamination of the cortical motor programming by noises that are ineffective or even disadvantageous for motor performance. Again, there is no significant change associated with movement in the spatial profiles of STN activities (Fig. 6f–i). The evident differences between control and parkinsonian animals in the spatiotemporal activity profiles in MC but not in STN lend further support to the deranged cortico-subthalamic information relay in PD.

### There are longer burst discharges but less responsiveness of STN to cortical input with dopaminergic deprivation

**Fig. 7:**
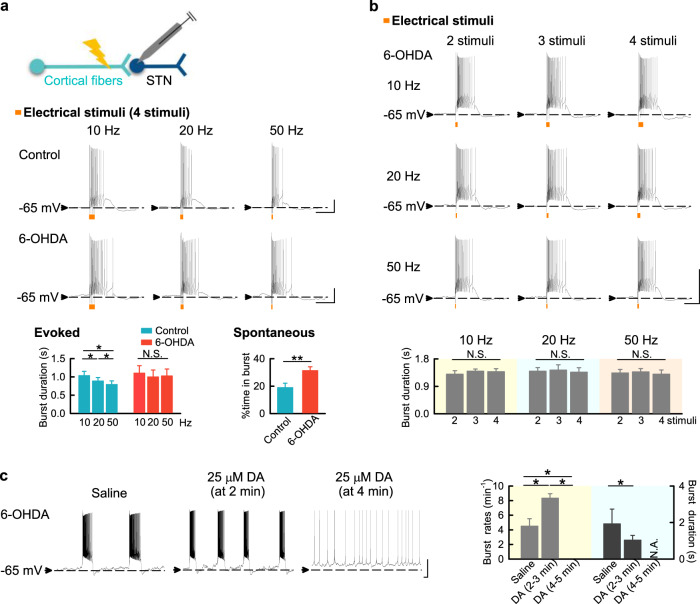
The relatively constant and longer STN bursts in response to MC activities in parkinsonian rats. **a** Four consecutive electrical stimuli at 10, 20, or 50 Hz on cortico-subthalamic fibers were applied to trigger burst discharges in STN neurons at ∼ −65 mV (“Evoked”). The burst duration gets shorter with higher frequency (and thus shorter duration) of stimuli in control (*n* = 8), but much less so in parkinsonian rats (6-OHDA) (*n* = 7). Also note that the mean burst duration is in general longer in parkinsonian than in control rats. In addition, the STN neurons from parkinsonian rats (*n* = 37) spend a larger proportion of time in spontaneous burst discharges than those from control rats (*n* = 15, with an evaluation time of 1 min in each neuron) (“Spontaneous”). **b** The burst duration in STN neurons from parkinsonian rats (*n* = 3–5) is very similar, whether the burst is elicited by 2, 3, or 4 stimuli given at 10, 20, or 50 Hz to the cortico-subthalamic fibers. **c** 25 μM dopamine shortens burst discharges in STN before a tonic or spike mode of discharges is finally achieved in ∼4 min (*n* = 5). The sample size denotes the number of neurons. Scale bars represent 1 s/20 mV. Data were presented as mean ± S.E.M. Data with two groups were analyzed with Mann–Whitney *U* test, and data with three groups were analyzed with Friedman tests (followed by the Wilcoxon signed-rank tests for further pairwise comparison). *P < 0.05, **P < 0.01, N.S., nonsignificant, N.A. indicates no bursts for analysis.

In view of the in vivo data suggesting dissociation between MC and STN activity in parkinsonian rats, we characterized STN responses to cortical drives from the hyperdirect pathway in brain slices. The generation of STN burst discharges relies heavily on hyperdirect cortico-subthalamic synaptic inputs^17^. We found that the burst duration in STN gets shorter with higher-frequency shorter-duration MC drives in control animals (Fig. 7a). In sharp contrast, the burst duration in STN tends to stay constant in response to different MC drives in parkinsonian rats, demonstrating a decrease in STN responsiveness to cortico-subthalamic input. The mean burst duration and total time spent in bursts are also longer in parkinsonian rats (Fig. 7a). The decrease in STN responsiveness is further illustrated in Fig. 7b, where different MC drives (at different frequencies and durations) all lead to the very similar duration of STN burst discharges. These parkinsonian features could be altered by supplementing dopamine. Before a tonic or spike mode of discharges is achieved, the burst duration in STN is transiently shortened upon application of dopamine in parkinsonian rats (Fig. 7c). This phenomenon is particularly discernible with relatively low concentration of dopamine (not shown), implying a dose-dependent modulation of STN burst length with transient local dopamine release. In the steady state, dopamine depolarizes the neuron and turns most burst discharges into the spike mode, presumably recapitulating the clinical effect of L-dopa or dopaminergic agonists on STN.

### Deep brain stimulation improves STN responsiveness to cortical input and partially rectifies MC pathological features in parkinsonian rats

**Fig. 8:**
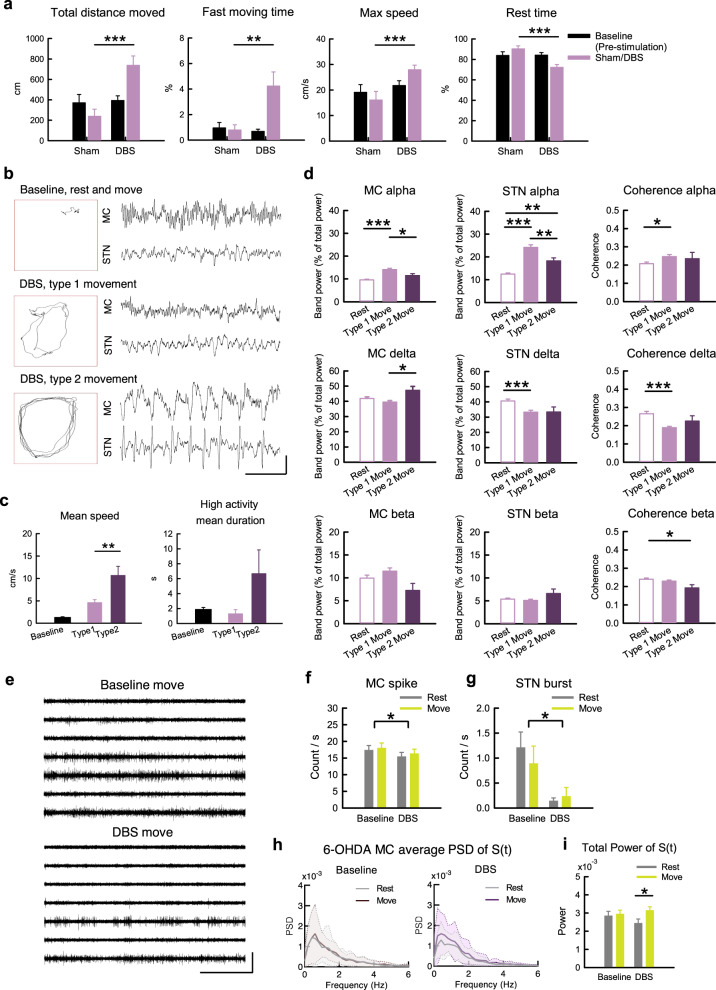
Electrophysiological changes with prokinetic effects of STN-DBS in parkinsonian rats. **a** Prokinetic effects of STN-DBS (depolarizing DC currents of −250 to −300μA) were examined in different parameters (*n* = 11 rats). **b** Sample maps of 30-s trajectories in OFT (boxes) and corresponding LFP recordings in MC and STN in a sample parkinsonian rat with baseline free running (including rest and movement), and type 1 and type 2 movements during DBS (Supplementary Movie 1 to 3). **c** Behavioral parameters in matched 30-s video recordings with type 1 and type 2 movements from the same OFT trial in parkinsonian rats. The “baseline” shows the data from sham stimulation in (**a**). *n* = 5 matched recordings from 3 rats. **d** Band power analysis according to the behavioral state during STN-DBS. *n* = 101, 76, and 14 10-s segments from 9 animals for rest, type 1 movement, and type 2 movement, respectively. **e** Sample sweeps of MU activities in MC from a parkinsonian rat during movement without (baseline) or with STN-DBS. **f**, **g** MC MU spike rates (**f**, *n* = 147 matches, i.e. 21 segments × 7 leads, from 7 animals) and STN bursts during STN-DBS (**g**, *n* = 5 matched segments from 4 leads from 2 animals). **h**, **i** Average PSD of S(t) (**h**) and the total power of S(t) (**i**) in MC (*n* = 37 at rest and *n* = 24 during movement in baseline, and *n* = 26 at rest and *n* = 34 during movement under STN-DBS, from 7 parkinsonian rats). PSD spectra were presented as mean (solid curves) ± standard deviation (shaded areas). Scale bars represent 1 s/500μV. Data in the bar plot were presented as mean ± S.E.M. Data were analyzed with ANCOVA (**a**), Mann–Whitney U tests (**c**), one-way ANOVA following Games–Howell post hoc tests (**d**), 2 × 2 dependent model ANOVA (**f**, **g**), and 2 × 2 independent model ANOVA (**i**). In (**f**) and (**g**), each horizontal line reports the simple main effect, and each square bracket reports the main effect of baseline vs. DBS in ANOVA. *P < 0.05, **p < 0.01, ***p < 0.001, N.S., nonsignificant.

**Fig. 9:**
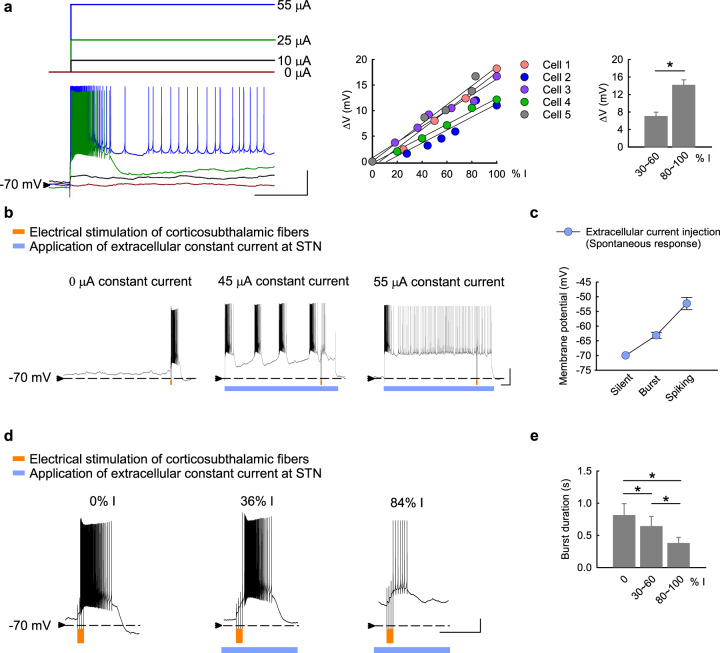
Modulation of membrane potential and discharge patterns of STN neurons by extracellularly applied constant currents. **a** The baseline membrane potential of STN neurons is changed correlative to the amplitude of extracellularly applied negative currents in acute brain slices from parkinsonian rats (left panel, *n* = 5). The percentage (% I) of the smallest current amplitude that switches the neuron into the spike mode of firing for each neuron is shown. There is tight correlation between the applied current amplitude and the change in membrane potential (∆*V*, middle and right panels). **b** Electrical stimulation of cortico-subthalamic fibers (orange marks, 4 pulses at 20 Hz) elicits subthalamic neuronal discharges, which are further modulated by the extracellular constant currents (blue bars). The 4-pulse stimulation elicits a burst of discharges outlasting the stimulation period in a STN neuron with a baseline membrane potential of ~−70 mV and without spontaneous activities (left panel). Application of an extracellular negative current of 45 μA modestly depolarizes the baseline membrane potential, at which spontaneous quasi-rhythmic burst discharges develop and the 4-pulse stimulation on cortical fibers elicits a shorter burst outlasting the stimulation period (middle panel). When a stronger extracellular negative current of 55 μA is given, the baseline membrane potential is even more depolarized and spontaneous spike discharges prevail. Here the same 4-pulse stimulation elicits no burst discharges but increases the spike frequency (only during but not outlasting the stimulation period, right panel). **c** The discharge pattern (electrical silence, spontaneously repetitive bursts, or spike-mode discharges) is closely correlated with the baseline membrane potential adjusted by extracellular constant currents (*n* = 4). **d** The duration of the burst discharges elicited by cortical fiber stimulation is the longest when no depolarizing extracellular currents are given (“0% I”), and gets shorter when stronger extracellular currents are given (e.g. “36 % I” vs. “84 % I”). **e** Cumulative results in (**d**) demonstrate a clear tendency of shorter burst duration with more depolarized baseline membrane potential upon extracellular current injection (*n* = 5–6). Scale bars represent 1 s/20 mV. Data with two groups were analyzed with Mann–Whitney *U* tests, and data with three groups were analyzed with Friedman tests (followed by the Wilcoxon signed-rank tests for further pairwise comparison). *P < 0.05.

**Fig. 10:**
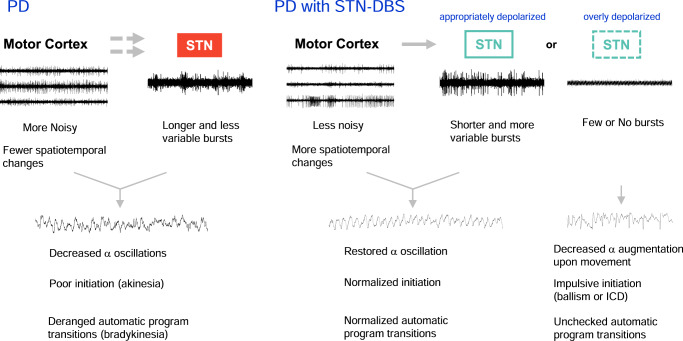
A summary of cortico-subthalamic motor control and mechanism of therapeutic effect of STN-DBS in PD. (Left) In PD, MC activities are increased, with fewer spatiotemporal changes upon movement. STN bursts are longer and less variable, and thus less responsive to different MC drives. There are consequently decreased α oscillations electrophysiologically and poor initiation (akinesia) as well as deranged automatic program transitions (bradykinesia) behaviorally. Here “akinesia” denotes difficulties in the initiation of both a movement and a non-motor cognitive process, and thus may include the deficiency of initiatives in PD. On the other hand, the impaired operations of the MC-STN (cortex-basal ganglia re-entrant loops) lead to deranged autonomic shifts of motor (and/or non-motor) programs and thus slowing the speed of execution of a set of movement (i.e. bradykinesia). It is of note that even in such a deranged system of PD, α augmentation upon movement is preserved, suggesting that α augmentation is an essential attribute of movement. (Right) In PD with STN-DBS, MC activities are decreased, with more spatiotemporal changes upon movement and restoration of α oscillations. STN bursts are decreased in number, and are shorter as well as more variable, and thus more responsive to different MC activities. However, if the STN is overly stimulated and depolarized, burst discharges would be excessively inhibited (few or no bursts). The principal role of STN burst discharges for an immediate check or “brake” for the cortical output is very much weakened. α oscillations are decreased again, with possibilities of impulsive initiation of a movement or even the other cognitive processes (e.g. ballism) and an unchecked automatic program transitions (propulsion or a movement without interim interruptions, such as the type 2 movement in Fig. 8).

Deep brain stimulation on STN (STN-DBS) is a well-established maneuver to ameliorate the motor symptoms in PD^61,62^. The behavior analysis in Fig. 8a clearly shows prokinetic effects of STN-DBS. With close observation of the animal behavior, we identified two distinct motor patterns with STN-DBS (Fig. 8b, Supplementary Video 1–3). One (type 1, Supplementary Video 2) is rather similar to normal movement, whereas the other one (type 2, Supplementary Video 3) is distinct from the normal presentation for the lack of interim interruptions. Moreover, the two types of behaviors are associated with different electrophysiological characteristics. Type 1 movement shows interim interruptions comparable to the movement in control animals, and is associated with an evident increase of α power in the MC-STN axis (Fig. 8c, d). In contrast, the α augmentation upon movement is less evident in type 2 movement despite the higher moving velocity (Fig. 8c, d). Because STN may serve a “brake” function in motor control, the type 1 and type 2 movement during STN-DBS presumably could be indicative of the presence of relatively normal motor gating and an overly suppressed brake function, respectively (see Discussion). It is of note that STN-DBS also rectifies the parkinsonian electrophysiological features in MC, including a decrease in overall MC spikes and an increase in spatiotemporal changes of MC discharges in addition to a decrease in STN burst discharges in situ (Fig. 8e–i). To inspect the cellular changes under DBS, extracellular constant currents were applied to STN in brain slices from parkinsonian rats (Fig. 9)^63^. The applied currents alter the membrane potential and consequently the discharge patterns of STN neurons in a dose (current amplitude)-dependent manner (Fig. 9a, b). For instance, either spontaneous burst discharges or burst discharges evoked by stimulation of cortico-subthalamic inputs can be shifted to spike discharges by application of sufficient extracellular constant current and thus strong depolarization of the neuron (Fig. 9b). Consistent with the findings with dopamine (Fig. 7c), the duration of STN burst discharges is shortened according to the amplitude of the applied currents and thus the extent of neuronal depolarization (Fig. 9d, e). It is plausible that either DBS or dopamine could depolarize STN neuron and improve their responsiveness to the cortex with shortened burst duration or shift to the spike mode of discharges. This may correspond to LFP attributes of increased α band power and α coherence in MC and STN with DBS. In view of the extracellular current amplitude-dependent changes in STN neuronal membrane potential and discharge pattern, the foregoing type 1 movement may correspond to a state with appropriate electrophysiological features in the MC-STN axis, whereas the type 2 movement may denote a state of DBS-induced excessive depolarization of STN neurons with inadequate burst discharges (Fig. 10). Continual and delicate tuning of the stimulation thus is highly advisable in clinical practice to maintain the optimal responsiveness of STN neurons by DBS considering the ever-changing local electrophysiological elements (see “Discussion”).

## Discussion

We have delineated the electrophysiological derangements in the MC-STN axis in parkinsonian animals, and the effect of STN-DBS on the derangements as well as correlative locomotor deficits with meticulous differentiation of the intervals of movement from rest and concomitant recordings of both LFP and MU electrophysiological activities. The MC and STN activities in parkinsonian rats are characterized by a decrease in overall α power in LFP (possibly replaced with an increase in β and δ power in MC and STN, respectively) (Fig. 1). Moreover, the locomotor activities are closely correlated with an increase in MC-STN α power and α coherence not only in control but also in parkinsonian animals (Figs. 2, 3, and Supplementary Fig. 2), suggesting a critical role of MC-STN collaborative activities in motor control. α oscillations were formerly thought to be a phasic change related to attentional function upon movement^37,64^ and were found to be associated with various motor tasks in the previous studies^33,48,49^. Because of the tight correlation between α augmentation and free movement (Figs. 2, 3), we would presume that α augmentation in the MC-STN axis is primarily associated with the movement itself rather than the other relevant mental processes. If the increase in α power upon movement corresponds to a concomitant phasic increase in dopamine release and consequent decrease in STN bursts (see below), then the lower MC α power at rest in parkinsonian animals (Figs. 2c, 3c) could implicate inadequate release of dopamine at rest (or more precisely, at the intention of movement) and derangement in the preparatory phase of cortical programming for future movements (i.e. deranged “preparatory potentials”)^65–67^. PD is therefore not just a movement disorder, but also a disorder “at rest” (well consistent with the MU findings, see below). In the meanwhile, the lower α power in STN but not in MC during movement in parkinsonian animals (Figs. 2c, 3c) may be consistent with a decrease in responsiveness of STN to cortico-subthalamic drives (Fig. 7, also see below). The tight association between α attenuation and the symptomatic pathogenesis of parkinsonian locomotor deficits is reminiscent of the lower-frequency oscillations in off-drug parkinsonian patients^68,69^. Moreover, θ-α oscillation in STN and in GPi was reported to be associated with motor performance in PD^49,70^. The correlation between α oscillations and locomotion thus could exist not only in the MC-STN axis, but also in the other key components of the cortico-subcortical re-entrant loops (the thalamocortico-basal ganglia circuitry). One limitation in the current study could be that we used recording electrodes with higher impedance for better extracellular neuronal (spike) recording. This may compromise the detection of LFP β augmentation in STN^71–73^. However, with a dataset with higher incidence of STN β augmentation using recording electrodes with lower impedance, changes in the α, but not β, power are still the closest associates with movement (Supplementary Fig. 1). The α attenuation in PD therefore could be a more consistent LFP consequence of dopaminergic deprivation (at least with the 6-OHDA hemiparkinsonian animal model) than β augmentation which is just variably correlated to the motor symptoms in PD.

With MU recordings, the overall MC activities (spike rates) normally do not increase from rest to movement but are characterized by more frequent changes in the spatiotemporal pattern (Fig. 6). We demonstrated that the overall MU spikes without sorting in MC are markedly increased in parkinsonian animals either at rest or during movement (Fig. 5), although it was inconsistently reported that the single-cell spike rates of corticostriatal neurons, corticofugal neurons, or layer V neurons in MC could be changed in parkinsonian animal models^74–78^. The enhanced MC activities in parkinsonian animals, however, fail to show more frequent changes upon movement (Figs. 5, 6). In addition, dopamine deprivation leads to more prevalent, longer, but less variable STN burst discharges in response to cortico-subthalamic drives. The increase in MU activities in the parkinsonian model may contribute to the changes in LFP, which is the spatiotemporal summation of local currents at every instant, and probably also contribute to the abnormalities in spatiotemporal pattern changes directly relevant to motor programmings in PD. The increase in MU activities is found only in MC, but not in STN (Fig. 5). This implicates that the noisy MC in PD is associated with a STN less faithfully following the cortical drives via the hyperdirect pathway. Specifically, a dopamine-deprived STN in PD is prone to burst discharges because of more hyperpolarized baseline membrane potentials (Figs. 7, 9)^79–82^. The burst duration is longer but less variable, and thus more time is allocated to burst discharges in STN in PD (Fig. 7). The more “fixed” and prolonged STN burst discharges would decrease the delicacy or faithfulness of STN responses to the incoming MC drives. The “built-in” feedback inhibition of premature MC activities via STN and GPi is then spatiotemporally less accurate, which may contribute to more cortical activities (noises) and thus self-sustaining vicious cycles^17,18^. This could embody the core of the deranged cortico-subthalamic transmission in PD or some types of parkinsonisms (e.g. the loss of MC entrainment of STN neurons reported for the parkinsonian features in late Huntington’s disease)^9–11,83–85^ (Fig. 10). Interestingly, excessive STN bursts and functional segregation of STN from MC are both remedied by either supplement of dopamine or STN-DBS (Figs. 7–9). These findings are consistent with the direct causal effect of excessive STN bursts in the locomotor deficits in parkinsonian animals^4–6,8^, and further support the imperativeness of phasic dopamine release upon the intention and especially the execution of movement. Like STN, substantia nigra pars compacta (SNpc) neurons show spike and burst mode of discharges^86^. The switch to burst discharges in SNpc neurons could be modulated by the glutamatergic innervations from the subthalamus and pedunculopontine nuclei^87,88^. If the burst discharges of STN would potentially inhibit an action, then the follow-up dopamine release may switch off the burst discharges in STN to release the “brake” for motor execution^18^. This may be the functional explanation why the midbrain dopaminergic neurons should be degenerated by ~80% for PD to become symptomatic^89,90^. In PD, the tonic level of dopamine is so low as to have excessive, prolonged, but less variable bursts which in turn fail to induce adequate and timely phasic dopamine increase. In this regard, the dopamine-deprived striatum, another major target of SNpc dopaminergic fibers, may have an impaired “filtering” function of MC activities and also contribute to the increase in MC activities. This is presumably ascribable to the weakened surrounding inhibition for ongoing programming (the “center-surrounded” model)^19,91^ due to deranged lateral inhibition between the medium spiny neurons in the striatopallidal direct and indirect pathways^92,93^. The impaired striatal filtering for MC activities, together with the relatively irresponsive STN with prolonged and less variable burst discharges, could lead to difficulties in motor execution (poor initiation) as well as imprecise derivations of consequential programs (bradykinesia) during movement in PD^18^. In this regard, there are many clinical situations which may share part of the behavioral manifestations of PD without specific degeneration of the midbrain dopaminergic neurons (“parkinsonisms”). It would be desirable to investigate the roles of more noisy MC and/or less responsive STN in different kinds of parkinsonisms, and design brain stimulation protocols based on more mechanistic rationales to achieve the best clinical effect.

It has been shown that excessive STN burst discharges have a direct causal relation with locomotor deficits in parkinsonian animals^4–6,8^. Also, simplistic hyperpolarization of STN neurons could turn a normal animal parkinsonian or ameliorate clinical hyperkinetic (choreiform or ballism-like) movement disorders, as if STN bursts have an inhibitory effect or serve as “brakes” on the locomotor behaviors^4,5,94^. STN-DBS may inactivate T-type Ca^2+^ and/or Na^+^ channels to decrease STN burst discharges and improve parkinsonian locomotor deficits^4,6^. In addition to a direct electrophysiological modulation of STN discharges in situ, we found that STN-DBS has an imperative network effect which is typified by a decrease in MU activities in MC in PD (Fig. 8). Consistently, high-frequency STN-DBS has also been reported to decrease MC activities in humans^95^. We provide a novel view on the enhanced locomotor activities by STN-DBS by a simplistic classification into two different types (Fig. 8). Type 1 shows interim interruptions (“brakes”) behaviorally, but type 2 is apparently abnormal, in terms of the restlessness or lack of interruptions of movement. Interestingly, only type 1 is associated with α augmentation upon movement but not type 2. In view of the tight correlation between STN burst discharges and the amplitude of extracellularly applied currents (Fig. 9), it is plausible that the apparently normal type 1 movement corresponds to a state of STN associated with an appropriate level of membrane depolarization and thus appropriate amount of burst discharges as well as α augmentation for motor execution. On the other hand, type 2 movement may correspond to a state of too much membrane depolarization and overinhibition or inadequacy of STN bursts. This is reminiscent of the clinical experiences that ballism-like movement or impulse control disorders (ICD) may develop with STN-DBS^96–98^. In this regard, it is of note that amelioration of ICD was also reported after STN-DBS^99,100^, a scenario probably confounded by concomitant use of dopaminergic or the other anti-PD medications^101–103^. We would propose that one should delicately balance the presumable primary “pro-ICD” effect of STN-DBS itself and the secondary”anti-ICD” effect of a decrease in dopaminergic medications with the advent of DBS. One therefore should continually and delicately tune the parameters of DBS in collaboration with the dopaminergic medications, keeping in mind the similarities and differences in the sites as well as the electrophysiological mechanisms of action and closely monitoring the behavioral consequences of both maneuvers.

In conclusion, we demonstrated a tight correlation between augmentation of α power in MC-STN and increase in locomotor activities in both normal and parkinsonian animals, and such “prokinetic” α activities are decreased in parkinsonian conditions. There are more MC activities (“noisy MC”) which may be correlated with β augmentation in parkinsonian animals. The noisy MC, however, is characterized by less spatiotemporal changes associated with movement. Together with the excessive, prolonged, but less variable STN bursts, this may well characterize a fundamental electrophysiological flaw in PD, namely a more “rigid” motor control system especially in the MC-STN axis. STN-DBS may at least partially restore the foregoing deficits, but should be meticulously tuned to avoid overstimulation and consequent adverse events.

## Methods

### 6-OHDA hemiparkinsonian rat model

The 6-OHDA-induced dopamine depletion models could be separated into two categories, bilateral lesions chiefly for the assessment of “non-motor/neuropsychiatric deficits”^58^ and unilateral lesions for the assessment of profound motor deficits^59^. SNc lesions could be more specific than MFB lesions, but the latter are more frequently applied probably chiefly because of the small size of SNc. It is relatively difficult to have a specific and complete lesion involving SNc (dopaminergic neuronal group A9) but leaving ventral tegmental areas (VTA, A10) untouched. In addition, the pathological findings in PD patients may not be limited to SNc^104–106^, and MFB model may results in motor deficits without neuropsychiatric deficits^60^. Therefore, to focus on parkinsonian motor deficits in this manuscript, we chose to take unilateral MFB lesion as the model. This model also has a well-established behavioral evaluation for a successful lesion (see below)^59^. Before surgery, male adult Wistar rats weighed 250–300 g were maintained under standard housing conditions at a constant temperature, humidity, and 12 h reversed light-dark cycle (light period from 8:00 am to 20:00 pm). A stainless steel cannula was inserted into the brain according to the coordinates of medial forebrain bundle (AP −2.4 mm, ML 2.2 mm, DV 8.0 mm from bregma)^107^. A total volume of 4 μl of 6-hydroxydopamine (6-OHDA) was slowly infused at a fixed velocity of 0.5 μl/min. Successful 6-OHDA hemiparkinsonian rat was defined as more than 25 contralateral rotations to the lesion side in 5 min after subcutaneous injection of apomorphine (0.05 mg/kg) on the 7^th^ day after surgery.

### Electrode implantation for in vivo electrophysiology recording

Arrays of 7 separated insulated tungsten microwires (diameter 0.0015 inch (38 μm), California Fine Wire Company) on a 10-pin connector (Omnetics Connector Corporation) were placed in layer V-VI of MC at AP 0 mm, ML 2 mm from bregma and DV 1–1.3 mm from cortical surface, and STN at AP −3.8 mm, ML 2.4 mm, and DV −7.5 to −7.8 from bregma ipsilateral to the 6-OHDA lesion side or right side in parkinsonian or control rat, respectively (Figs. 1–6, S1, and S2). For extracellular recording during STN-DBS, the recording electrode in STN was re-located 0.3 mm anterior to the stimulation electrode (Fig. 8, also see below). The depth of electrode at STN was also confirmed by the characteristic firing patterns of STN during the procedure.

### Electrode implantation for deep brain stimulation

We used two-channel electrode systems with insulated stainless steel electrode (diameter 0.008 inch (203 μm), Plastics One) for deep brain stimulation. One electrode was placed into STN at AP −3.8 mm, ML 2.4 mm, and DV −7.5 to −7.8 from bregma ipsilateral to the 6-OHDA lesion side. The other electrode was connected to a stainless steel skew on the skull as the ground electrode. To achieve simultaneous stimulation and recording at STN, recording tungsten microwires were pre-fixed at 0.3 mm anterior to the stimulation electrode before implantation.

### In vivo electrophysiology recording

Multi- as well as single-unit and local field potential (LFP) recordings were documented in free-running rats implanted with microwire electrodes (during the open field test). Electric signals were obtained with a headstage connected to rat’s head and wired to an amplifier (Model 3600, A-M system), with total 100× amplification and 3000 Hz low-pass filter, and digitized with an analog-to-digital converter (Datawave Technology) at 25000 Hz sampling rate. The signals were further band-pass filtered into 0–100 Hz for LFP and 300–3000 Hz for multi-unit activities offline with a digital IIR filter (Sciworks 10.0, Datawave Technology).

### Open field test (OFT)

The open-field arena was constructed of a black Plexiglas box with 45 × 45 cm floor and 25 cm wall, and surrounded by a black-out cloth and a Faraday cage to achieve a dark, noise-free environment. The control or hemiparkinsonian rat was introduced to the open field test after being well-acquainted with the experimental environment. Each trial included a 5-min concurrent behavior video and electrophysiology recordings. Each subject would have 4 trials, and all trials were performed in the evening upon light-dark transition time, with an intertrial interval of at least 24 h to avoid carry-over effect. Total movement distance, movement trajectory, speed, mobile time, and global activity in each epoch were analyzed by a video-tracking system software (Smart 3.0, Panlab Harvard Apparatus). High activity states are defined by pixels change detected in Smart video-tracking system, with the following thresholds: <30 cm^2^/s for immobility, >700 cm^2^/s for high activity). In STN-DBS studies (Fig. 8), each trial of open field test included four sequential 5-min epochs of recording. After the first epoch of recording for baseline free movement, 5.5-min −250 μA or −300 μA direct current (DC) deep brain stimulation, or sham stimulation was applied. The second epoch of recording started after initial 30 s of stimulation to avoid shock behaviors induced by the initial voltage change of electric stimulation. After stimulation, two subsequent epochs were recorded to evaluate the post-stimulation behavior. The motor patterns without (baseline) or with STN-DBS were identified by offline inspection on the video recordings. The enhancement of motor activities by DBS is classified into type 1 movements, which have interim interruptions comparable to that in normal conditions (Supplementary Video 2), and type 2 movements, which have essentially no interruptions (defined as continuous walking around the open field box without direction change or interruption more than 3 s by other movement such as rearing or grooming for 30 s or longer, Supplementary Video 3). In Fig. 8c, matched 30-s video recordings with type 1 and type 2 movement were included for analysis of behavioral parameters.

### Local field potential (LFP) analysis

Recordings for local field potential analysis were down-sampling to 500 Hz by Sciworks software. Power spectral density (PSD) of LFP was derived from fast Fourier transform of the recordings using Welch’s method with hanning window, 1/2 overlap, and 0.5-Hz frequency resolution in MATLAB (*pwelch* function from signal processing toolbox, MATLAB R2018b, MathWorks). Average band power in delta (1–4 Hz), alpha (7–10 Hz), and beta (20–40 Hz) band were computed by integration of the PSD estimates and the corresponding frequencies. PSD and band power were then normalized to the total power (1–250 Hz) and presented as the percentages of total to avoid influences by the variations of total power during the recording. Frequency below 1 Hz were excluded due to the low-frequency artifacts during movement. Magnitude-squared coherence between MC and STN in each frequency band was then computed for LFP coherence analysis (*mscohere* function from signal processing toolbox, MATLAB R2018b, MathWorks).

### Analysis of baseline LFP and LFP correlative to the behavioral states in control and parkinsonian rats

The “baseline” LFP does not discriminate the behavioral state (i.e. no differentiation between rest and moving), and all of the 5-min recordings without contaminations by high-voltage spindles and movement or mechanical artifacts from OFT trials were included for baseline LFP analysis. High-voltage spindles were recognized as a generalized high-amplitude (more than ± 2 mV) spike-and-wave pattern at 5–10 Hz and with an abrupt onset and end^108,109^. Behaviorally, the rat was motionless or “absence-like”. The average of the data from all of the eligible leads in MC or STN was taken as the mean LFP value for each 5-min recording. The parallel average total movement distance recorded by video-tracking systems for all of the included 5-min recordings reaches significant difference between control and parkinsonian group (control: 713.0 ± 134.9 cm, *n* = 29; parkinsonian: 423.9 ± 97.6 cm, *n* = 20, p = 0.002, Student’s *t* test). For the comparison between the electrophysiological findings at rest and during the moving state, the recordings were further divided into 10-s segments according to the behavioral state defined by the moving velocity (moving velocity >4 cm/s and <2.5 cm/s for the moving and resting states, respectively). The segments were also confirmed by visual examination of the video recordings to assure a continuous rest or moving state. Only the segments without change in behavior for 10 s were included. These segments are further classified with additional criteria for multi-unit analysis (see below). The final average velocities of the included segments at rest are 1.38 cm/s and 1.3 cm/s, and of the segments during moving 8.42 cm/s and 6.78 cm/s, in control and parkinsonian rats, respectively. For LFP analysis, data from all eligible leads in MC or STN were averaged for each included segment. Supplementary Fig. 3 summarizes the inclusion criteria for electrophysiological analysis according to the behavioral state.

### Correlation analysis between LFP and moving velocity

To demonstrate temporal change of LFP, we calculated the power and coherence spectral density of the 5-min baseline LFP recordings in 10-s steps with a shift in 1 s between two consecutive steps to construct a series of LFP data in 1-s bins, using the same method based on fast Fourier transformation as above-mentioned. The PSD and band power of each bin were then plotted against elapsed time to make the time-frequency spectrogram and time-dependent plot of band power, respectively. Time-dependent change of coherence spectral density and band coherence were plotted in the same way. On the other hand, instantaneous velocity was calculated from the coordinate change of rat’s center of mass in each video frame at 16 fps captured by the Smart video-tracking system. The time series of instantaneous velocity were further average within the 10-s steps, with 1-s shift to make the to make the time-velocity analysis similar to that described above for spectrograms. Cross-covariance between the two plots (time-frequency analysis and time-velocity analysis) was calculated by MATLAB function *xcov*, and the correlation coefficient at zero time lag were documented (e.g. in the box plot in Figs. 2, 3, and Supplementary Fig. 2).

### Multi-unit analysis

Only the 10-s segments with an unchanged behavioral (rest or movement) state were included for further analysis. To avoid contamination with muscle or mechanical activities artifacts during free movement, and high-voltage spindles at rest, we further inspected all recordings to reject unqualified recordings for multi-unit analysis. The movement or mechanical artefacts were typically recognized: (1) waveforms with amplitude larger than ± 2 mV synchronously occurred in all electrodes in MC or STN, (2) waveforms with similar morphology synchronously occurring in all electrodes in MC or STN tightly associated with rat behaviors (e.g. bruxing, grooming, or licking), (3) known environmental artifacts marked during experiment. The inclusion criteria for electrophysiological analysis according to the behavioral states are described in Supplementary Fig. 3. In vivo extracellular recordings were processed offline by a spike detection software (Sciworks 10.0, Datawave Technology). We set the spike detection threshold at 4x of the estimate median absolute deviation of signals^110^,

1 $$\sigma \;\left( {\sigma = \frac{{{{{\mathrm{median}}}}\left( {|{{{\mathrm{signals}}}}|} \right)}}{{0.6745}}} \right)$$

All detects were included as spikes in multi-unit profiles of each electrode in each segment. In Fig. 5f, g, bin spike rate change were calculated by the absolute change of spike rate between adjacent 1-s bins, normalized with average spike rate of each 10-s segment, and described as

2 $$\frac{{\mathop {\sum }\nolimits_{t = 1}^{n - 1} \left| {{{{\mathrm{Bin}}}}\;{{{\mathrm{spike}}}}\;{{{\mathrm{rate}}}}_t - {{{\mathrm{Bin}}}}\;{{{\mathrm{spike}}}}\;{{{\mathrm{rate}}}}_{t + 1}} \right|}}{{(n - 1)\mathop {\sum }\nolimits_{t = 1}^n {{{\mathrm{Bin}}}}\;{{{\mathrm{spike}}}}\;{{{\mathrm{rate}}}}_t/n}},\;{{{\mathrm{n}}}} = 10$$

In Fig. 8g, STN bursts were analyzed under the burst definition: 4 or more consecutive spikes with interspike interval <20 ms assessed by interspike interval algorithm^8^.

### Spike spatial dissimilarity analysis

SPIKE-distance (S), a time-resolved measure of spike train dissimilarity, of spike trains in MC or STN in each 10-s segments was obtained by SPIKY, a MATLAB-based program developed by Kreuz et al.^111^. To avoid the effect of variation in spike rate among 7 leads in SPIKE-distance calculation, only the leads with spike rate >5 Hz were included and only the segments with >5 included leads were included for dissimilarity analysis. To perform frequency spectrum analysis of temporal changes of SPIKE-distance, the extracted SPIKE-distance (S) from SPIKY was re-sampled to forming a 500-Hz time series of data with interpolation method and the average were set to zero. The frequency spectrum of temporal changes of SPIKE-distance were calculated by fast Fourier transform using Welch’s method with hanning window, 1/2 overlap, and 0.2-Hz frequency resolution in MATLAB. The total power of temporal changes of SPIKE-distance was computed by integrating all the PSD estimates and their corresponding frequencies.

### Histological verification of the implanted electrodes

Rats were anesthetized with overdosed urethane (2 g/kg i.p., Sigma-Aldrich) and then transcardially perfused with 4% paraformaldehyde in PBS. The brain was removed and immersed in 4% paraformaldehyde for 8 h, and dehydrated with 20% sucrose at 4 °C for 2 days. Coronal sections were sliced into the thickness of 30 μm at MC, STN, SNc and striatum by a frozen microtome. Sections at MC and STN were stained with crystal violet (Nissl stain) to confirm the location of the recording electrode. The sections containing SNc and striatum were washed with PBS, followed by a suppression procedure in 5% fetal bovine serum in 0.1% Triton, and incubated with anti-tyrosine hydroxylase antibody (dilution 1:500, Sigma-Aldrich) overnight at 4 °C, followed by secondary fluorescent antibody (goat anti-rabbit IgG (H + L) highly cross-adsorbed secondary antibody, dilution 1:200, Invitrogen). Images were taken using fluorescence microscope (Zeiss Axiomager, M1). Data were collected only from the animals with confirmed electrode location and dopamine depletion by histological examinations (Supplementary Fig. 4).

### Brain slice preparation and in vitro electrophysiological recording

Brain slices that contain the cortico-subthalamic networks^8,17,112^ were prepared from normal or 6-OHDA-lesioned Wistar rats (aged postnatal days of 23–61). Briefly, the whole brain was quickly removed and immersed into a cold oxygenated cutting solution (in mM, containing 87 NaCl, 37.5 choline chloride, 25 NaHCO_3_, 25 glucose, 2.5 KCl, 1.25 NaH_2_PO_4_, 7 MgCl_2_, and 0.5 CaCl_2_). Parasagittal brain slices (250–300 μm thick) were then cut on a vibratome (Leica VT1200S; Leica, Nussloch). The slices were incubated in the oxygenated cutting solution for 25 min at 30 °C and then transferred into an oxygenated saline for 25 min at 30 °C (in mM, containing 125 NaCl, 26 NaHCO_3_, 25 glucose, 2.5 KCl, 1.25 NaH_2_PO_4_, 1 MgCl_2_, and 2 CaCl_2_) before electrophysiological recordings. For recording, a slice was put in a chamber with stable perfusion of oxygenated (95% O_2_/5% CO_2_) saline with a constant flow rate of ∼5 ml/min maintained by a peristaltic pump (Gilson Medical Electric). Whole-cell current-clamp recordings from STN neurons (visualized with a 60x water immersion objective on the microscope Olympus BX51WI) were obtained using glass pipettes (3–5 MΩ) containing the following internal solution (in mM): 116 KMeSO_4_, 6 KCl, 2 NaCl, 20 HEPES, 0.5 EGTA, 4 MgATP, 0.3NaGTP, 10 NaPO_4_creatine, and pH 7.25 adjusted with KOH. The presynaptic fibers were stimulated using a pair of glass electrodes filled with saline and placed on the internal capsule in the position between the striatum and globus pallidus and on the ventral side of STN, through which the cortical fibers travel to innervate the STN^17,63^. The fibers from the thalamus which are located on the dorsal side of STN shall be hardly stimulated with such a configuration^63,112–115^. Stimulus intensities were adjusted to determine the optimal stimulation (usually ∼0.3 mA with pulse-width of 0.3–0.5 ms) and delivered by a stimulus isolator (World Precision Instruments A356,). For application of extracellular constant currents, a pair of stimulating electrodes filled with saline was placed near the recorded STN neurons. Recordings were acquired with an Multiclamp 700B amplifier (MDS Analytical Technologies) filtered at 1 kHz and digitized at 10–20 kHz with a Digidata-1440 analog/digital interface (MDS Analytical Technologies). Stock solutions of different pharmacological agents (all purchased from Sigma Aldrich) were obtained by dissolving the drugs in DMSO or distilled water, stored at −20 °C, and diluted into the bath reservoir immediately before application. All analyses were based on the averaged data from a 60 s of stable continuous recording. Data were excluded if the spike overshoot was below 0 mV. Burst discharge is defined as having at least 3 consecutive spikes within an interval shorter than 100 ms. Burst duration is measured as the time interval between the peak of the first spike and that of the last spike.

### Statistics

Numerical data and statistical analyses were managed with SigmaPlot (Systat Software), Microsoft Excel (Microsoft), and SPSS 19.0 (IBM). In Fig. 1, Supplementary Fig. 1a and d, and Supplementary Fig. 2a, Mann–Whitney *U* tests were applied for comparing LFP between control and 6-OHDA group. For the study in the control vs. 6-OHDA and rest vs. move two factor structures, we applied 2 × 2 factorial ANOVA and simple main effect tests (Figs. 2–6, and 8f, g, and i). For LFP and MU analyses (Figs. 2–5), mixed models were applied for pairs of rest and movement conditions in both control and. 6-OHDA groups. For dissimilarity analyses (Figs. 6, 8i), independent models were applied. For analyses in STN-DBS study (Fig. 8f and g), dependent models were applied for matched rest and movement in baseline and during DBS. The main effects, interaction effects, and simple main effects from 2 × 2 factorial ANOVA in all relevant figures are listed in an additional supplementary table (Supplementary Table 1). In Fig. 8a, we applied analysis of covariance (ANCOVA) to evaluate the effect of DBS on behavioral variables in open field tests to have a control of the baseline (pre-stimulation) behavioral variables. Data in Fig. 8a without homogeneity in regression coefficients were tested with the Johnson-Neyman method^116^. In Fig. 8c, we applied one-way ANOVA with *post hoc* tests with the Games–Howell method. Data in Fig. 8c not passed Levene’s equal variance tests were tested by Welch’s F tests. For data from in vitro electrophysiology recording (Figs. 7, 9), data with two groups were analyzed with Mann–Whitney *U* tests, and data with three groups were analyzed with Friedman tests (followed by the Wilcoxon signed-rank tests for further pairwise comparison). The statistical analysis results are reported in Supplementary Table 1. Scatter plots for individual data from the dataset of bar graphs in which *n* < 10 are shown in Supplementary Fig. 5.

### Study approval

The study and all procedures were approved by the Institutional Animal Care and Use Committee of National Taiwan University College of Medicine and College of Public Health, and that of Chang Gung University, Taiwan.

## Supplementary information

Supplementary Information

Supplementary Video 1

Supplementary Video 2

Supplementary Video 3

## Data Availability

The data that support the findings of this study are available in the paper including supplementary materials or from the corresponding author upon reasonable request.

## Code availability

The MATLAB codes for data analysis used in this article of this study are available from the corresponding author upon reasonable request.

## References

[1] Hollerman JR, Grace AA (1992). Subthalamic nucleus cell firing in the 6-OHDA-treated rat: basal activity and response to haloperidol. Brain Res..

[2] Bergman H, Wichmann T, Karmon B, DeLong MR (1994). The primate subthalamic nucleus. II. Neuronal activity in the MPTP model of parkinsonism. J. Neurophysiol..

[3] Benazzouz A (2002). Intraoperative microrecordings of the subthalamic nucleus in Parkinson’s disease. Mov. Disord..

[4] Tai CH (2012). Subthalamic discharges as a causal determinant of parkinsonian motor deficits. Ann. Neurol..

[5] Tai C-H, Yang Y-C, Pan M-K, Huang C-S, Kuo C-C (2011). Modulation of subthalamic T-type Ca2+ channels remedies locomotor deficits in a rat model of Parkinson disease. J. Clin. Invest..

[6] Yang YC, Tai CH, Pan MK, Kuo CC (2014). The T-type calcium channel as a new therapeutic target for Parkinson’s disease. Pflug. Arch..

[7] Pan MK (2016). Neuronal firing patterns outweigh circuitry oscillations in parkinsonian motor control. J. Clin. Invest.

[8] Huang C-S, Wang G-H, Tai C-H, Hu C-C, Yang Y-C (2017). Antiarrhythmics cure brain arrhythmia: The imperativeness of subthalamic ERG K+ channels in parkinsonian discharges. Sci. Adv..

[9] Chu H-Y, McIver EL, Kovaleski RF, Atherton JF, Bevan MD (2017). Loss of hyperdirect pathway cortico-subthalamic inputs following degeneration of midbrain dopamine neurons. Neuron.

[10] Wang YY (2018). Impaired glutamatergic projection from the motor cortex to the subthalamic nucleus in 6-hydroxydopamine-lesioned hemi-parkinsonian rats. Exp. Neurol..

[11] Mathai A (2015). Reduced cortical innervation of the subthalamic nucleus in MPTP-treated parkinsonian monkeys. Brain.

[12] Baudrexel S (2011). Resting state fMRI reveals increased subthalamic nucleus-motor cortex connectivity in Parkinson’s disease. NeuroImage.

[13] Kurani AS (2015). Subthalamic nucleus–sensorimotor cortex functional connectivity in de novo and moderate Parkinson’s disease. Neurobiol. Aging.

[14] Jia Q, Gao L, Zhang J, Wu T, Chan P (2018). Altered functional connectivity of the subthalamic nucleus during self-initiated movement in Parkinson’s disease. J. Neuroradiol..

[15] Chen HM, Sha ZQ, Ma HZ, He Y, Feng T (2018). Effective network of deep brain stimulation of subthalamic nucleus with bimodal positron emission tomography/functional magnetic resonance imaging in Parkinson’s disease. CNS Neurosci. Ther..

[16] Pan MK (2014). Deranged NMDAergic cortico-subthalamic transmission underlies parkinsonian motor deficits. J. Clin. Invest.

[17] Huang C-S (2021). Conveyance of cortical pacing for parkinsonian tremor-like hyperkinetic behavior by subthalamic dysrhythmia. Cell Rep..

[18] Lee LN (2021). An electrophysiological perspective on Parkinson’s disease: symptomatic pathogenesis and therapeutic approaches. J. Biomed. Sci..

[19] Nambu A, Tokuno H, Takada M (2002). Functional significance of the cortico-subthalamo-pallidal ‘hyperdirect’ pathway. Neurosci. Res..

[20] Mink JW (2003). The Basal Ganglia and involuntary movements: impaired inhibition of competing motor patterns. Arch. Neurol..

[21] Nambu A (2005). A new approach to understand the pathophysiology of Parkinson’s disease. J. Neurol..

[22] Brown P (2001). Dopamine dependency of oscillations between subthalamic nucleus and pallidum in Parkinson’s disease. J. Neurosci..

[23] Levy R (2002). Dependence of subthalamic nucleus oscillations on movement and dopamine in Parkinson’s disease. Brain.

[24] Sharott A (2005). Dopamine depletion increases the power and coherence of beta-oscillations in the cerebral cortex and subthalamic nucleus of the awake rat. Eur. J. Neurosci..

[25] Mallet N (2008). Disrupted dopamine transmission and the emergence of exaggerated beta oscillations in subthalamic nucleus and cerebral cortex. J. Neurosci..

[26] Kuhn AA (2009). Pathological synchronisation in the subthalamic nucleus of patients with Parkinson’s disease relates to both bradykinesia and rigidity. Exp. Neurol..

[27] Kuhn AA, Kupsch A, Schneider GH, Brown P (2006). Reduction in subthalamic 8-35 Hz oscillatory activity correlates with clinical improvement in Parkinson’s disease. Eur. J. Neurosci..

[28] López-Azcárate J (2010). Coupling between beta and high-frequency activity in the human subthalamic nucleus may be a pathophysiological mechanism in Parkinson’s disease. J. Neurosci..

[29] Kuhn AA (2004). Event-related beta desynchronization in human subthalamic nucleus correlates with motor performance. Brain.

[30] Talakoub O (2016). Time-course of coherence in the human basal ganglia during voluntary movements. Sci. Rep..

[31] Crowell AL (2012). Oscillations in sensorimotor cortex in movement disorders: an electrocorticography study. Brain.

[32] Weinberger M (2006). Beta oscillatory activity in the subthalamic nucleus and its relation to dopaminergic response in Parkinson’s disease. J. Neurophysiol..

[33] Singh A, Levin J, Mehrkens JH, Botzel K (2011). Alpha frequency modulation in the human basal ganglia is dependent on motor task. Eur. J. Neurosci..

[34] Ray NJ (2008). Local field potential beta activity in the subthalamic nucleus of patients with Parkinson’s disease is associated with improvements in bradykinesia after dopamine and deep brain stimulation. Exp. Neurol..

[35] Chen CC (2010). Complexity of subthalamic 13-35 Hz oscillatory activity directly correlates with clinical impairment in patients with Parkinson’s disease. Exp. Neurol..

[36] Heinrichs-Graham E (2014). Neuromagnetic evidence of abnormal movement-related beta desynchronization in Parkinson’s disease. Cereb. Cortex.

[37] Brittain J-S, Brown P (2014). Oscillations and the basal ganglia: motor control and beyond. NeuroImage.

[38] Choi JW (2020). Altered pallidocortical low-beta oscillations during self-initiated movements in Parkinson disease. Front Syst. Neurosci..

[39] Fischer P (2018). Alternating modulation of subthalamic nucleus beta oscillations during stepping. J. Neurosci..

[40] Bichsel O (2018). Functionally separated networks for self-paced and externally-cued motor execution in Parkinson’s disease: Evidence from deep brain recordings in humans. NeuroImage.

[41] Leventhal DK (2012). Basal ganglia beta oscillations accompany cue utilization. Neuron.

[42] Abbasi O (2018). Unilateral deep brain stimulation suppresses alpha and beta oscillations in sensorimotor cortices. NeuroImage.

[43] Kuhn AA (2008). High-frequency stimulation of the subthalamic nucleus suppresses oscillatory beta activity in patients with Parkinson’s disease in parallel with improvement in motor performance. J. Neurosci..

[44] Blumenfeld Z (2015). Sixty hertz neurostimulation amplifies subthalamic neural synchrony in Parkinson’s disease. PLoS One.

[45] Foffani G (2006). Subthalamic oscillatory activities at beta or higher frequency do not change after high-frequency DBS in Parkinson’s disease. Brain Res Bull..

[46] Rossi L (2008). Subthalamic local field potential oscillations during ongoing deep brain stimulation in Parkinson’s disease. Brain Res Bull..

[47] Alonso-Frech F (2006). Slow oscillatory activity and levodopa-induced dyskinesias in Parkinson’s disease. Brain.

[48] Wojtecki L, Elben S, Vesper J, Schnitzler A (2017). The rhythm of the executive gate of speech: subthalamic low-frequency oscillations increase during verbal generation. Eur. J. Neurosci..

[49] Tan H (2013). Frequency specific activity in subthalamic nucleus correlates with hand bradykinesia in Parkinson’s disease. Exp. Neurol..

[50] Giannicola G (2013). The effects of levodopa and deep brain stimulation on subthalamic local field low-frequency oscillations in Parkinson’s disease. Neurosignals.

[51] Eusebio A (2009). Resonance in subthalamo-cortical circuits in Parkinson’s disease. Brain.

[52] Walker HC (2012). Short latency activation of cortex during clinically effective subthalamic deep brain stimulation for Parkinson’s disease. Mov. Disord..

[53] Jech R (2006). Deep brain stimulation of the subthalamic nucleus affects resting EEG and visual evoked potentials in Parkinson’s disease. Clin. Neurophysiol..

[54] Devos D (2004). Subthalamic nucleus stimulation modulates motor cortex oscillatory activity in Parkinson’s disease. Brain.

[55] Swann N (2011). Deep brain stimulation of the subthalamic nucleus alters the cortical profile of response inhibition in the beta frequency band: a scalp EEG study in Parkinson’s disease. J. Neurosci..

[56] Cavanagh JF (2011). Subthalamic nucleus stimulation reverses mediofrontal influence over decision threshold. Nat. Neurosci..

[57] Kibleur A, David O (2018). Electroencephalographic read-outs of the modulation of cortical network activity by deep brain stimulation. Bioelectron. Med..

[58] Magnard R (2016). What can rodent models tell us about apathy and associated neuropsychiatric symptoms in Parkinson’s disease?. Transl. Psychiatry.

[59] Deumens R, Blokland A, Prickaerts J (2002). Modeling Parkinson’s disease in rats: an evaluation of 6-OHDA lesions of the nigrostriatal pathway. Exp. Neurol..

[60] Delaville C (2012). Emerging dysfunctions consequent to combined monoaminergic depletions in Parkinsonism. Neurobiol. Dis..

[61] Deuschl G (2006). A randomized trial of deep-brain stimulation for Parkinson’s disease. N. Engl. J. Med..

[62] Follett KA (2010). Pallidal versus subthalamic deep-brain stimulation for Parkinson’s disease. N. Engl. J. Med..

[63] Hamani, C. et al. Subthalamic nucleus deep brain stimulation: basic concepts and novel perspectives. *eNeuro* **4**, 10.1523/eneuro.0140-17.2017 (2017). 10.1523/ENEURO.0140-17.2017 PMC5617209 28966978

[64] Litvak V (2010). Resting oscillatory cortico-subthalamic connectivity in patients with Parkinson’s disease. Brain.

[65] Dick JP (1989). The Bereitschaftspotential is abnormal in Parkinson’s disease. Brain.

[66] Touge T, Werhahn KJ, Rothwell JC, Marsden CD (1995). Movement-related cortical potentials preceding repetitive and random-choice hand movements in Parkinson’s disease. Ann. Neurol..

[67] Cunnington R, Iansek R, Johnson KA, Bradshaw JL (1997). Movement-related potentials in Parkinson’s disease. Motor imagery and movement preparation. Brain.

[68] Stoffers D (2007). Slowing of oscillatory brain activity is a stable characteristic of Parkinson’s disease without dementia. Brain.

[69] Vardy AN (2011). Slowing of M1 activity in Parkinson’s disease during rest and movement–an MEG study. Clin. Neurophysiol..

[70] Silberstein P (2003). Patterning of globus pallidus local field potentials differs between Parkinson’s disease and dystonia. Brain.

[71] Kita, Y. et al. Three-micrometer-diameter needle electrode with an amplifier for extracellular in vivo recordings. *Proc Natl Acad Sci USA* **118**, 10.1073/pnas.2008233118 (2021). 10.1073/pnas.2008233118 PMC8072214 33846241

[72] Miceli, S., Ness, T. V., Einevoll, G. T. & Schubert, D. Impedance spectrum in cortical tissue: implications for propagation of lfp signals on the microscopic level. *eNeuro* **4**, 10.1523/eneuro.0291-16.2016 (2017). 10.1523/ENEURO.0291-16.2016 PMC5282548 28197543

[73] Nelson MJ, Pouget P (2010). Do electrode properties create a problem in interpreting local field potential recordings?. J. Neurophysiol..

[74] Li Q (2012). Therapeutic deep brain stimulation in Parkinsonian rats directly influences motor cortex. Neuron.

[75] Pasquereau B, DeLong MR, Turner RS (2016). Primary motor cortex of the parkinsonian monkey: altered encoding of active movement. Brain.

[76] Goldberg JA (2002). Enhanced synchrony among primary motor cortex neurons in the 1-methyl-4-phenyl-1,2,3,6-tetrahydropyridine primate model of Parkinson’s disease. J. Neurosci..

[77] Wang J (2017). Network-wide oscillations in the parkinsonian state: alterations in neuronal activities occur in the premotor cortex in parkinsonian nonhuman primates. J. Neurophysiol..

[78] Hyland BI, Seeger-Armbruster S, Smither RA, Parr-Brownlie LC (2019). Altered recruitment of motor cortex neuronal activity during the grasping phase of skilled reaching in a chronic rat model of unilateral parkinsonism. J. Neurosci..

[79] Beurrier C, Congar P, Bioulac B, Hammond C (1999). Subthalamic nucleus neurons switch from single-spike activity to burst-firing mode. J. Neurosci..

[80] Bevan MD, Wilson CJ (1999). Mechanisms underlying spontaneous oscillation and rhythmic firing in rat subthalamic neurons. J. Neurosci..

[81] Bevan MD, Magill PJ, Hallworth NE, Bolam JP, Wilson CJ (2002). Regulation of the timing and pattern of action potential generation in rat subthalamic neurons in vitro by GABA-A IPSPs. J. Neurophysiol..

[82] Cragg SJ, Baufreton J, Xue Y, Bolam JP, Bevan MD (2004). Synaptic release of dopamine in the subthalamic nucleus. Eur. J. Neurosci..

[83] Kita H, Kita T (2011). Cortical stimulation evokes abnormal responses in the dopamine-depleted rat basal ganglia. J. Neurosci..

[84] Isaacs BR (2019). Cortico-basal white matter alterations occurring in Parkinson’s disease. PLoS ONE.

[85] Callahan JW, Abercrombie ED (2015). Age-dependent alterations in the cortical entrainment of subthalamic nucleus neurons in the YAC128 mouse model of Huntington’s disease. Neurobiol. Dis..

[86] Goto Y, Otani S, Grace AA (2007). The Yin and Yang of dopamine release: a new perspective. Neuropharmacology.

[87] Lee CR, Tepper JM (2009). Basal ganglia control of substantia nigra dopaminergic neurons. J. Neural Transm. Suppl.

[88] Quik M, Wonnacott S (2011). α6β2* and α4β2* nicotinic acetylcholine receptors as drug targets for Parkinson’s disease. Pharm. Rev..

[89] Chang JW, Wachtel SR, Young D, Kang UJ (1999). Biochemical and anatomical characterization of forepaw adjusting steps in rat models of Parkinson’s disease: studies on medial forebrain bundle and striatal lesions. Neuroscience.

[90] Hefti F, Melamed E, Wurtman RJ (1980). Partial lesions of the dopaminergic nigrostriatal system in rat brain: biochemical characterization. Brain Res..

[91] Mink JW (1996). The basal ganglia: focused selection and inhibition of competing motor programs. Prog. Neurobiol..

[92] Taverna S, Ilijic E, Surmeier DJ (2008). Recurrent collateral connections of striatal medium spiny neurons are disrupted in models of Parkinson’s disease. J. Neurosci..

[93] Tecuapetla F, Koós T, Tepper JM, Kabbani N, Yeckel MF (2009). Differential dopaminergic modulation of neostriatal synaptic connections of striatopallidal axon collaterals. J. Neurosci..

[94] Tai C-H, Pan M-K, Tseng S-H, Wang T-R, Kuo C-C (2020). Hyperpolarization of the subthalamic nucleus alleviates hyperkinetic movement disorders. Sci. Rep..

[95] Ren L (2020). Subthalamic nucleus stimulation modulates motor epileptic activity in humans. Ann. Neurol..

[96] Hälbig TD (2009). Subthalamic deep brain stimulation and impulse control in Parkinson’s disease. Eur. J. Neurol..

[97] Smeding HM (2007). Pathological gambling after bilateral subthalamic nucleus stimulation in Parkinson disease. J. Neurol. Neurosurg. Psychiatry.

[98] Lim SY (2009). Dopamine dysregulation syndrome, impulse control disorders and punding after deep brain stimulation surgery for Parkinson’s disease. J. Clin. Neurosci..

[99] Abbes M (2018). Subthalamic stimulation and neuropsychiatric symptoms in Parkinson’s disease: results from a long-term follow-up cohort study. J. Neurol. Neurosurg. Psychiatry.

[100] Lhommée E (2012). Subthalamic stimulation in Parkinson’s disease: restoring the balance of motivated behaviours. Brain.

[101] Corvol JC (2018). Longitudinal analysis of impulse control disorders in Parkinson disease. Neurology.

[102] Weintraub D (2010). Impulse control disorders in Parkinson disease: a cross-sectional study of 3090 patients. Arch. Neurol..

[103] Weintraub D (2010). Amantadine use associated with impulse control disorders in Parkinson disease in cross-sectional study. Ann. Neurol..

[104] McRitchie DA, Cartwright HR, Halliday GM (1997). Specific A10 dopaminergic nuclei in the midbrain degenerate in Parkinson’s disease. Exp. Neurol..

[105] Del Tredici, K. & Braak, H. In *Madame Curie Bioscience Database [Internet]*, https://www.ncbi.nlm.nih.gov/books/NBK6077/ (Landes Bioscience, Austin, 2000).

[106] Braak H, Del Tredici K (2009). Neuroanatomy and pathology of sporadic Parkinson’s disease. Adv. Anat. Embryol. Cell Biol..

[107] Paxinos, G. & Watson, C. *The Rat Brain in Stereotaxic Coordinates*. 6th edn (Academic Press, 2007).

[108] Dejean C, Gross CE, Bioulac B, Boraud T (2007). Synchronous high-voltage spindles in the cortex-basal ganglia network of awake and unrestrained rats. Eur. J. Neurosci..

[109] Berke JD, Okatan M, Skurski J, Eichenbaum HB (2004). Oscillatory entrainment of striatal neurons in freely moving rats. Neuron.

[110] Rey HG, Pedreira C, Quian Quiroga R (2015). Past, present and future of spike sorting techniques. Brain Res Bull..

[111] Kreuz T, Mulansky M, Bozanic N (2015). SPIKY: a graphical user interface for monitoring spike train synchrony. J. Neurophysiol..

[112] Beurrier C, Ben-Ari Y, Hammond C (2006). Preservation of the direct and indirect pathways in an in vitro preparation of the mouse basal ganglia. Neuroscience.

[113] Parent A, Hazrati LN (1995). Functional anatomy of the basal ganglia. II. The place of subthalamic nucleus and external pallidum in basal ganglia circuitry. Brain ResBrain ResRev..

[114] Hamani C, Saint-Cyr JA, Fraser J, Kaplitt M, Lozano AM (2004). The subthalamic nucleus in the context of movement disorders. Brain.

[115] Lein ES (2007). Genome-wide atlas of gene expression in the adult mouse brain. Nature.

[116] Tu, C.-T. *Experimental Design and ANCOVA*. (Wu-Nan Culture Enterprise, 2017).

