# Supplementary material for: Deep brain stimulation rectifies the noisy cortex and irresponsive subthalamus to improve parkinsonian locomotor activities
Source: NPJ Parkinsons Dis. 2022 Jun 20;8:77. doi: 10.1038/s41531-022-00343-6 (PMC9209473; doi:10.1038/s41531-022-00343-6)
Supplement: Supplementary file 1 — Supplementary Information [file 41531_2022_343_MOESM1_ESM.pdf]

Supplementary Information

for

Deep Brain Stimulation Rectifies the Noisy Cortex and Irresponsive Subthalamus to Improve Parkinsonian Locomotor Activities.

Lan-Hsin Nancy Lee, Chen-Syuan Huang, Ren-Wei Wang, Hsing-Jung Lai, Chih-Ching Chung, Ya-Chin Yang, and Chung-Chin Kuo

Supplementary Figure 1

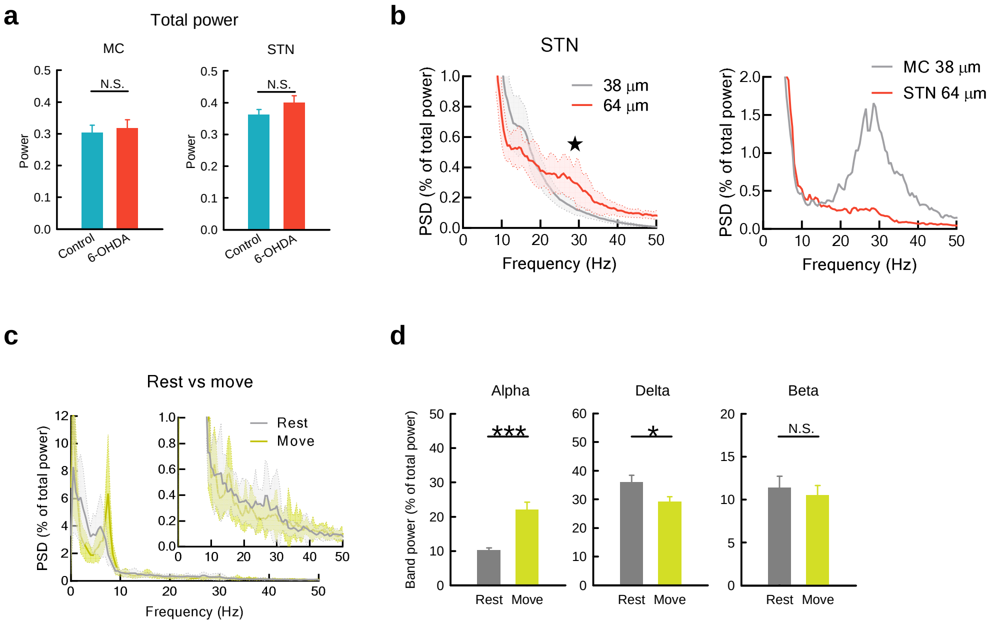

Supplementary Figure 1. The total power LFP analysis and recording with thicker electrodes
a There is no difference in the total power from 0-250 Hz LFP spectral analysis of 5-minute recordings in open field tests (OFT) between control and parkinsonian (6-OHDA) rats in both MC and STN. The analyses are from the same recordings in Fig. 1 (n = 29 recordings from 10 control rats, n = 20 recordings from 11 parkinsonian rats). **b** Left: Average PSD spectra of LFP recordings in STN using 38-μm (data from Fig. 1) or 64-μm electrodes from 20 and 9 parkinsonian rats in OFT, respectively. There is a better detection of the β peak in STN with thicker electrodes (star mark). Right: PSD spectra of a 2-minute LFP recording in OFT shows a more prominent β peak in MC than in STN from a parkinsonian rat where thicker (64 μm) electrodes are implanted in STN. **c** Average PSD spectra in STN recorded with 64 μm electrodes show a marked increase of α but not β power from rest to movement in parkinsonian rats (n = 14 segments during 10-second continuous rest or movement periods from 9 rats). **d** The band power analysis from part **c** reveals a significant increase of the α power and decrease of the δ power from rest to movement in parkinsonian rats. The β band power shows no difference from rest to movement. The results are similar to those obtained with 38 μm recording electrodes in Fig. 1. PSD spectra are presented as mean (solid curves) ± standard deviation (shaded areas). Data in bar graphs are presented as mean ± S.E.M. and analyzed with Mann-Whitney U tests. *$p < 0.05$, ***$p < 0.001$, N.S., nonsignificant.

Supplementary Figure 2

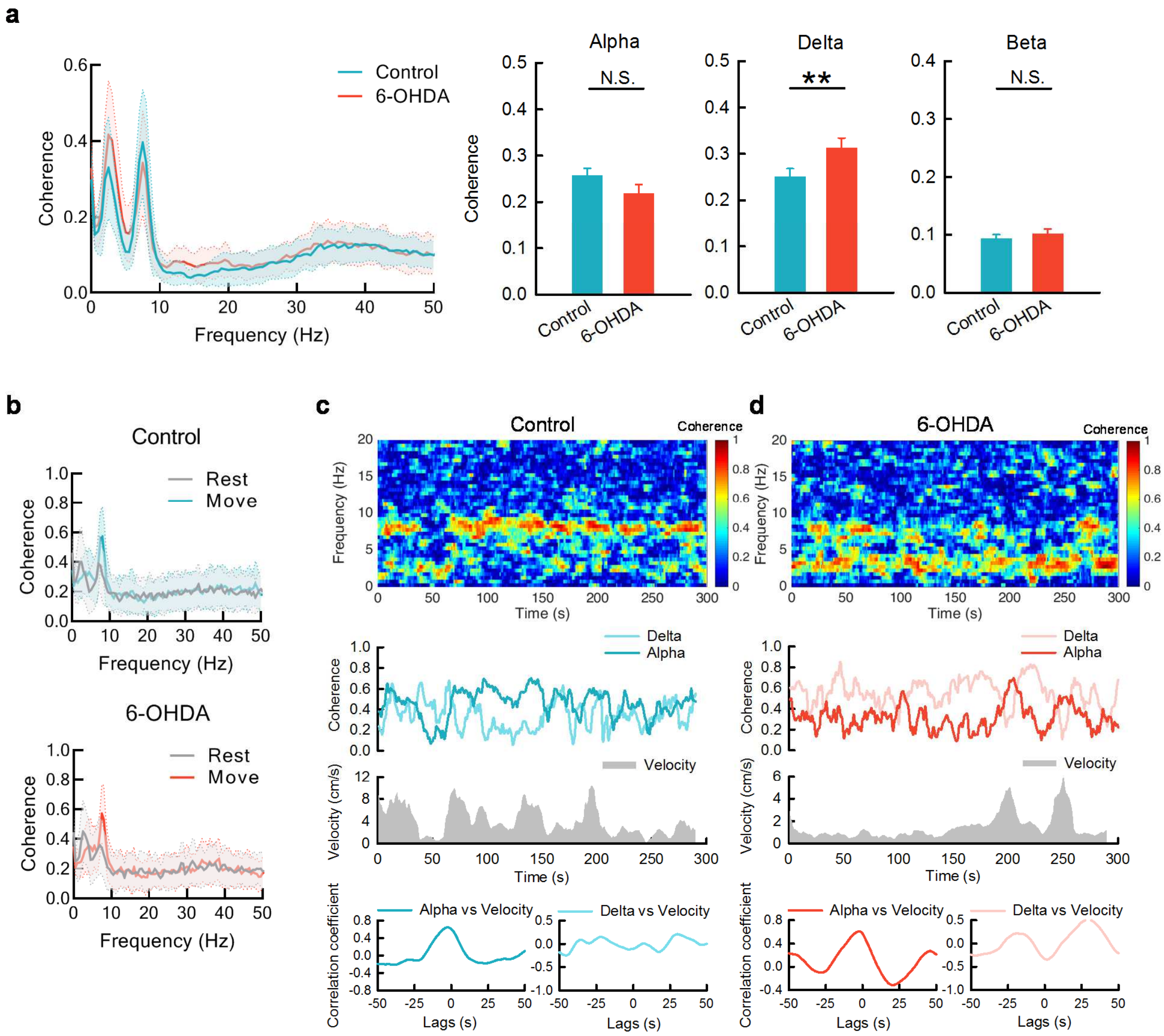

Supplementary Figure 2. The analysis of cortico-subthalamic coherence in control and parkinsonian rats.

a The average coherence spectra from continuous 5-minute LFP recordings reveal slightly higher MC-STN coherence in α frequencies and significantly lower coherence in δ frequencies in control than in parkinsonian (6-OHDA) rats. The coherence in β frequencies shows little difference. n = 29 and 20 recordings from 10 control and 11 parkinsonian (6-OHDA) rats, respectively. **b** The average MC-STN coherence spectra show a marked increase of MC-STN α coherence from rest to movement in both control (blue) and parkinsonian rats (red). Data are collected from the same rats in Fig. 2 and 3. n = 52 and 50 matched segments of rest and movement from 10 control and 11 parkinsonian rats, respectively. **c, d** Representative coherence spectrograms (top) and the changes of α coherence (middle, upper) reveal a trend of positive correlation between α coherence and moving velocity in OFT (grey areas, middle, lower) in a control (**c**) and a parkinsonian (**d**) rats. Cross-covariance analysis between time-varying α coherence and moving velocity (bottom) also reveals a peak of correlation coefficient at zero time lag in both control and parkinsonian rats. There is little or just a low-level correlation between δ coherence and moving velocity in both cases. PSD spectra are presented as mean (solid curves) ± standard deviation (shaded areas). Data in bar graphs are presented as mean ± S.E.M. and analyzed with Mann-Whitney U tests. **p <0.01, N.S., nonsignificant.

Supplementary Figure 3

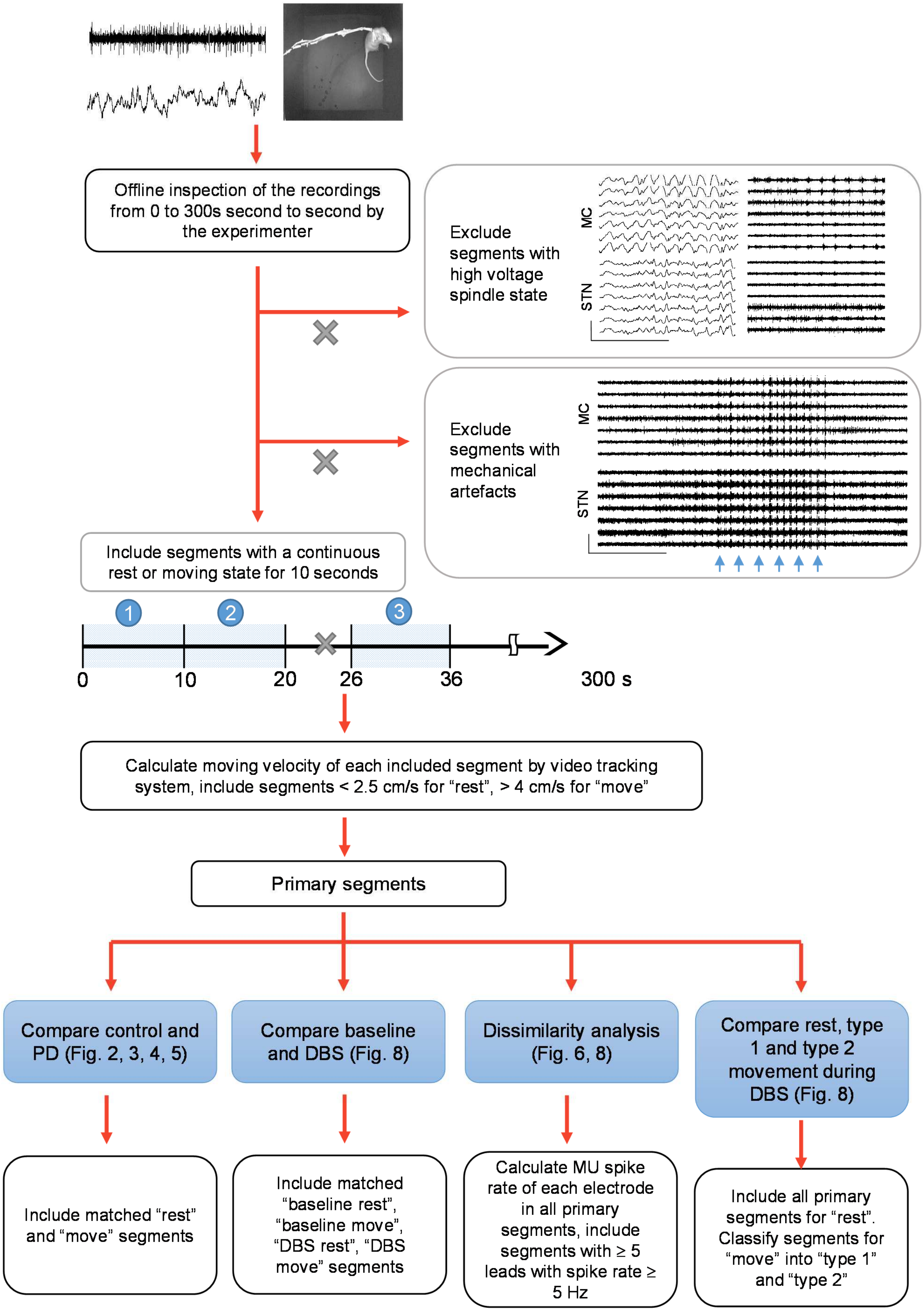

Supplementary Figure 3. Inclusion criteria for electrophysiological analysis according to behavioral states.

The offline-analyzed LFP, multi-unit spikes, and video recordings from each 5-minute OFT were inspected simultaneously second to second by an experimenter. Segments with 10-seconds of continuous rest or movement in video recordings without high voltage spindles or mechanical artefacts were included. States of high voltage spindles (HVS) were electrophysiologically recognized as a generalized high-amplitude (more than ± 2 mV) spike-and-wave pattern at 5-10 Hz in LFP, with concomitant generalized bursts at 5-10 Hz in spike recordings and with an abrupt onset and end. Behaviorally, the rat was motionless or “absence-like” in the video. The movement or mechanical artifacts (blue arrows) were typically recognized by the criteria detailed in Methods. The segments were included from the beginning of recording by each 10 seconds, until HVS, artefacts, or discontinuation of the behavioral state were encountered. The moving velocity of collected segments was calculated by the video tracking system, and segments with velocity < 2.5 cm/s for rest and > 4 cm/s for movement were included as primary segments for further analysis. For LFP and MU analysis comparing control and 6-OHDA (Figs. 2, 3, 4, and 5), matched rest and movement segments were included in chronological order. For MC MU and STN burst analysis in STN-DBS study (Fig. 8, f and g), matched rest and movement in baseline and DBS were included in reversed chronological order. For dissimilarity analysis (Figs. 6 and 8, i), all primary segments with ≥ 5 leads with MU spike rate ≥ 5 Hz were included. For LFP analysis comparing different movement states during STN-DBS (Fig. 8d), primary segments for movement were further classified into type 1 and type 2 movement according to the video criteria of type 2 movement (see Methods). Scale bars represent 1s/500μV.

Supplementary Figure 4

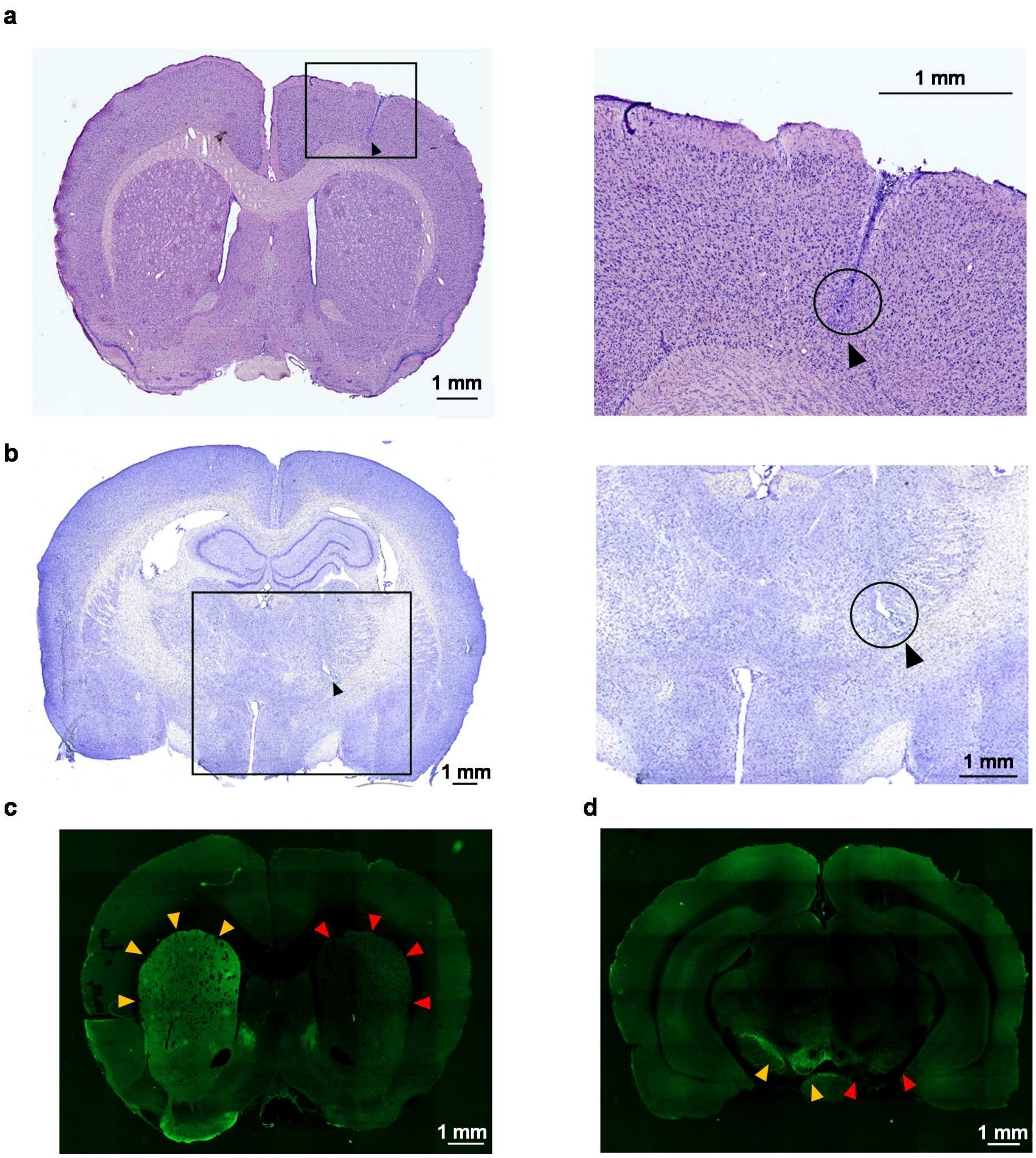

Supplementary Figure 4. Histological examination of the location of recording electrodes and dopamine depletion by 6-OHDA.

a, b The locations of the recording electrode tips (indicated by black arrow heads) in MC and STN are confirmed by coronal section of rat brains with crystal violet (Nissl) staining. The right panel is zoomed from the black box in the left panel to show the locations. **c, d** Images of tyrosine hydroxylase immunohistochemistry from a rat with unilateral 6-OHDA lesion. There is unilateral loss of dopaminergic innervation in the ipsilateral striatum (**c**, red arrow heads) and the substantia nigra pars compacta and the ventral tegmental area (**d**, red arrow heads).

Supplementary Figure 5

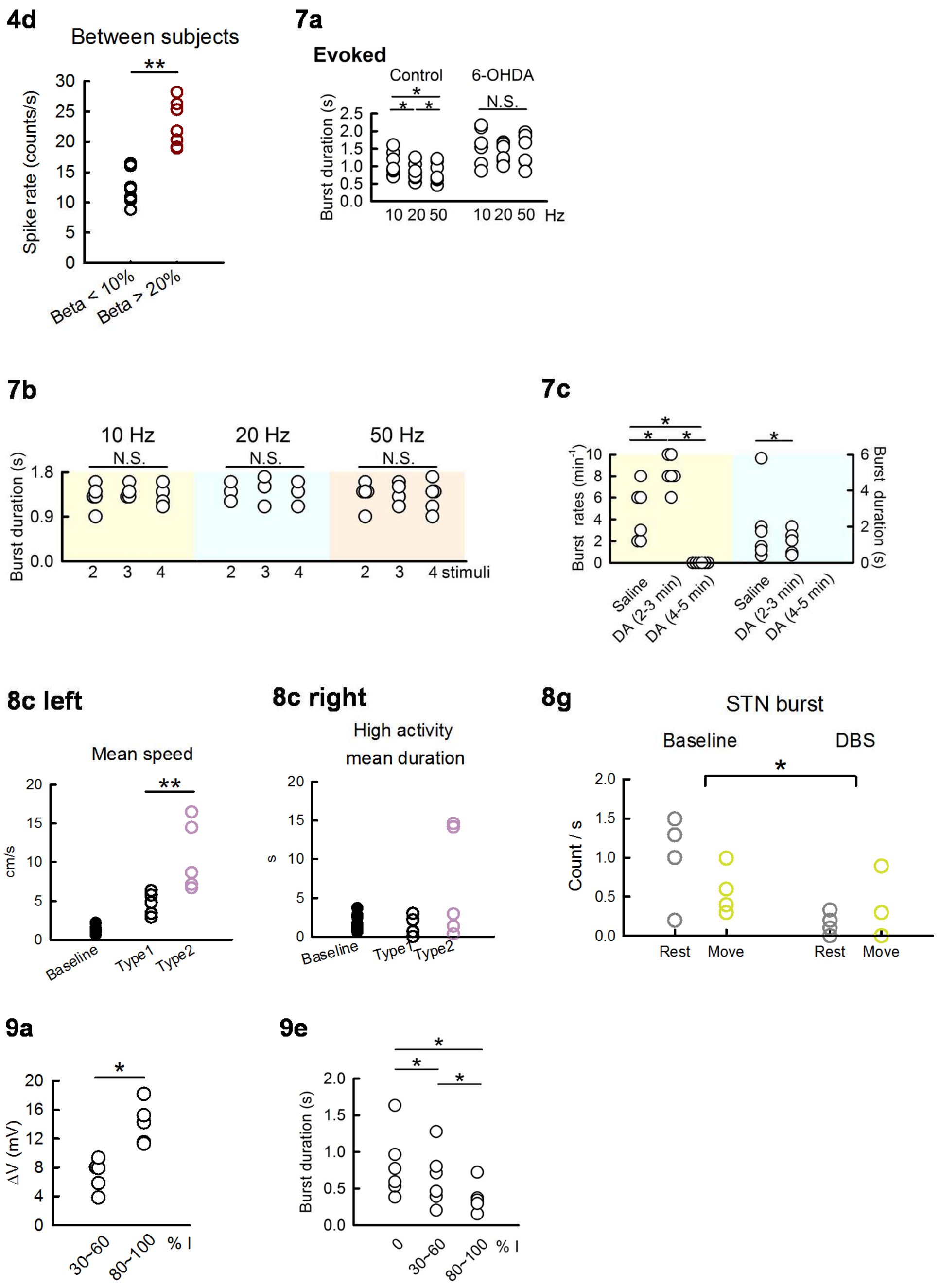

Supplementary Figure 5. Scatter plots for individual data from the dataset of bar graphs in which n < 10.

Supplementary Table 1. Reports of the statistical analysis results.

Fig. 1: Mann-Whitney U test

	Variables	P (2-tailed)
Control vs. 6-OHDA	MC alpha	.002
	MC delta	.640
	MC beta	.064
	STN alpha	.009
	STN delta	.063
	STN beta	.823

Fig. 2c: 2 x 2 mixed model ANOVA

	Main effect	df	F	P	Simple main effect	F	P
MC alpha	Control vs 6-OHDA (Between-subjects)	1	5.695	.019	Rest	8.070	.005
					Move	.691	.407
	Rest vs Move (within-subject)	1	153.096	.000	Control	59.490	.000
					6-OHDA	95.376	.000
	Interaction	1	2.474	.119			
	Error	100					
MC delta	Control vs 6-OHDA (Between-subjects)	1	2.128	.148	Rest	.074	.787
					Move	4.483	.035
	Rest vs Move (within-subject)	1	25.639	.000	Control	6.090	.014
					6-OHDA	21.826	.000
	Interaction	1	2.585	.111			
	Error	100					

Fig. 3c, 3h: 2 x 2 mixed model ANOVA

	Main effect	df	F	P	Simple main effect	F	P
STN alpha	Control vs 6-OHDA (Between-subjects)	1	12.058	.001	Rest	3.605	.059
					Move	11.082	.001
	Rest vs Move (within-subject)	1	519.595	.000	Control	290.850	.000
					6-OHDA	231.096	.000
	Interaction	1	1.180	.280			
	Error	100					
STN delta	Control vs 6-OHDA (Between-subjects)	1	10.638	.002	Rest	9.592	.002
					Move	3.953	.048
	Rest vs Move (within-subject)	1	96.546	.000	Control	40.766	.000
					6-OHDA	56.260	.000
	Interaction	1	.784	.378			
	Error	100					
Coherence alpha	Control vs 6-OHDA (Between-subjects)	1	1.354	.247	Rest	1.334	.249
					Move	.363	.547
	Rest vs Move (within-subject)	1	98.913	.000	Control	46.263	.000
					6-OHDA	52.702	.000
	Interaction	1	.177	.674			
	Error	100					
Coherence delta	Control vs 6-OHDA (Between-subjects)	1	.102	.751	Rest	1.730	.190
					Move	.588	.444
	Rest vs Move (within-subject)	1	38.047	.000	Control	8.685	.004
					6-OHDA	33.052	.000
	Interaction	1	4.168	.044			
	Error	100					

Fig. 4a: 2 x 2 mixed model ANOVA

	Main effect	df	F	P	Simple main effect	F	P
MC beta	Control vs 6-OHDA (Between-subjects)	1	18.665	.000	Rest	14.126	.000
					Move	20.588	.000
	Rest vs Move (within-subject)	1	.612	.436	Control	0.190	.663
					6-OHDA	2.321	.129
	Interaction	1	1.940	.167			
	Error	100					
STN beta	Control vs 6-OHDA (Between-subjects)	1	.196	.659	Rest	1.724	.191
					Move	.303	.583
	Rest vs Move (within-subject)	1	15.957	.000	Control	17.287	.000
					6-OHDA	2.303	.131
	Interaction	1	3.340	.071			
	Error	100					
Coherence beta	Control vs 6-OHDA (Between-subjects)	1	.103	.749	Rest	.001	.974
					Move	.290	.591
	Rest vs Move (within-subject)	1	.444	.507	Control	.006	.938
					6-OHDA	.734	.392
	Interaction	1	.311	.578			
	Error	100					

Fig. 4d, left: Wilcoxon signed rank test

	Variables	P (2-tailed)
Low beta vs. High beta	MC MU Spike rate	.039

Fig. 4d, right: Mann-Whitney U test

	Variables	P (2-tailed)
Beta < 10% vs. Beta > 20%	MC MU Spike rate	.001

Fig. 5b, d, f, h: 2 x 2 mixed model ANOVA

	Main effect	df	F	P	Simple main effect	F	P
MC MU spike	Control vs 6-OHDA (Between-subjects)	1	64.750	.000	Rest	57.956	.000
					Move	66.578	.000
	Rest vs Move (within-subject)	1	8.081	.005	Control	9.074	.003
					6-OHDA	1.056	.304
	Interaction	1	1.891	.169			
	Error	712					
STN MU spike	Control vs 6-OHDA (Between-subjects)	1	.510	.475	Rest	1.916	.166
					Move	.000	.989
	Rest vs Move (within-subject)	1	10.269	.001	Control	0.270	.604
					6-OHDA	15.831	.000
	Interaction	1	6.137	.013			
	Error	712					
MC MU temporal change	Control vs 6-OHDA (Between-subjects)	1	17.402	.000	Rest	2.885	.092
					Move	25.699	.000
	Rest vs Move (within-subject)	1	34.686	.000	Control	38.471	.000
					6-OHDA	4.522	.035
	Interaction	1	8.307	.005			
	Error	68					
STN MU	Control vs 6-OHDA	1	1.140	.291	Rest	4.058	.047

temporal change	(Between-subjects)				Move	.127	.722
	Rest vs Move (within-subject)	1	1.090	.301	Control	0.326	.569
					6-OHDA	4.637	.034
	Interaction	1	3.541	.066			
	Error	50					

Fig. 6e, i: 2 x 2 independent model ANOVA

	Main effect	df	F	P	Simple main effect	F	P
MC time average of S	Control vs 6-OHDA	1	.134	.715	Rest	.074	.786
					Move	.061	.806
	Rest vs Move	1	11.977	.001	Control	5.027	.027
					6-OHDA	7.380	.008
	Interaction	1	.000	.991			
	Error	118					
MC total power of S(t)	Control vs 6-OHDA	1	8.490	.004	Rest	1.543	.217
					Move	8.123	.005
	Rest vs Move	1	8.714	.004	Control	7.247	.008
					6-OHDA	1.903	.170
	Interaction	1	1.413	.237			
	Error	118					
STN time average of S	Control vs 6-OHDA	1	.418	.519	Rest	.166	.684
					Move	1.728	.191
	Rest vs Move	1	.011	.916	Control	1.125	.291
					6-OHDA	.510	.477
	Interaction	1	1.490	.225			
	Error	106					
STN total power of S(t)	Control vs 6-OHDA	1	5.241	.024	Rest	3.767	.055
					Move	1.688	.197
	Rest vs Move	1	3.826	.053	Control	3.685	.058
					6-OHDA	.938	.335
	Interaction	1					
	Error	106					

Fig. 7a, Evoked: Friedman tests

	Variables	P (2-tailed)
Control<br>10 Hz vs 20 Hz vs 50 Hz	Burst duration (s)	.000
6-OHDA<br>10 Hz vs 20 Hz vs 50 Hz	Burst duration (s)	.206

Fig. 7a, Evoked: Wilcoxon signed rank test

	Variables	P (2-tailed)
Control<br>10 Hz vs. 20 Hz	Burst duration (s)	.012
Control<br>20 Hz vs. 50 Hz	Burst duration (s)	.011
Control<br>10 Hz vs. 50 Hz	Burst duration (s)	.012

Fig. 7a, Spontaneous: Mann-Whitney U test

	Variables	P (2-tailed)
Control vs 6-OHDA	%time in burst	.009

Fig. 7b: Friedman tests

	Variables	P (2-tailed)
10 Hz stimuli<br>2 vs 3 vs 4	Burst duration (s)	.717
20 Hz stimuli<br>2 vs 3 vs 4	Burst duration (s)	.368
50 Hz stimuli<br>2 vs 3 vs 4	Burst duration (s)	.779

Fig. 7c, left: Friedman tests

	Variables	P (2-tailed)
Saline vs DA (2-3 min) vs DA (4-5 min)	Burst rate (min^{-1})	.003

Fig. 7c: Wilcoxon signed rank test

	Variables	P (2-tailed)
Saline vs DA (2-3 min)	Burst rate (min^{-1})	.043
DA (2-3 min) vs DA (4-5 min)	Burst rate (min^{-1})	.026
Saline vs DA (4-5 min)	Burst rate (min^{-1})	.027
Saline vs DA (2-3 min)	Burst duration (s)	.046

Fig. 8a: ANCOVA

Dependent variable	Covariate	df	Error	Behavioral variables	F	P
**DBS**	Sham	1	19	Total distance	30.277	.000
				Fast moving time	10.407	.004
				Max speed	15.514[#]	.000
				Rest time	43.520	.000
						[#]**Johnson-Neyman method**

Fig. 8c: Mann-Whitney U test

	Behavioral variables	P (2-tailed)
Type1 vs Type 2	Mean speed	.008
	High activity mean duration	.310

Fig. 8d: one-way ANOVA

Dependent variables	df	Error	F	P	Games-Howell *post hoc* test	P	95% Confidence Interval	
							Lower Bound	Upper Bound
MC alpha	1	19	23.826*	.000	Rest-Type1	.000	-6.0977	-2.9629
					Rest-Type2	.089	-4.1242	.2588
					Type1-Type2	.036	.1493	5.0458
STN alpha	1	19	47.073*	.000	Rest-Type1	.000	-14.6630	-8.7138
					Rest-Type2	.002	-9.3318	-2.2845
					Type1-Type2	.004	1.6834	10.0770
Coherence alpha	1	19	3.633	.028	Rest-Type1	.020	-.0723	-.0051
					Rest-Type2	.721	-.1202	.0648
					Type1-Type2	.951	-.0824	.1043
MC delta	1	19	3.638	.028	Rest-Type1	.278	-1.2768	5.9899
					Rest-Type2	.144	-12.4742	1.5488
					Type1-Type2	.026	-14.7667	-.8717

STN delta	1	19	9.287	.000	Rest-Type1	.000	3.2056	11.6046
					Rest-Type2	.131	-1.8396	16.3003
					Type1-Type2	.999	-9.3175	8.9679
Coherence delta	1	19	14.244*	.000	Rest-Type1	.000	.0427	.1102
					Rest-Type2	.420	-.0400	.1216
					Type1-Type2	.479	-.1139	.0426
MC beta	1	19	3.079	.048	Rest-Type1	.251	-3.7658	.7321
					Rest-Type2	.264	-1.5564	6.9512
					Type1-Type2	.054	-.0602	8.4888
STN beta	1	19	2.409	.093	Rest-Type1	.661	-.5070	1.0947
					Rest-Type2	.486	-3.8574	1.4646
					Type1-Type2	.344	-4.1689	1.1884
Coherence beta	1	19	4.117	.018	Rest-Type1	.440	-.0100	.0314
					Rest-Type2	.047	.0005	.0947
					Type1-Type2	.139	-.0102	.0840
								***Welch's F test**

Fig. 8f, g: 2 x 2 dependent model ANOVA

	Main effect	df	F	P	Simple main effect	F	P
MC MU spike rate	Baseline vs DBS	1	6.674	.011	Rest	1.082	.299
					Move	.817	.367
	Rest vs Move	1	2.218	.139	Baseline	.113	.737
					DBS	.224	.636
	Interaction	1	.082	.775			
	Error	146					
	Error (all)	587					
STN burst rate	Baseline vs DBS	1	12.278	.025	Rest	9.196	.008
					Move	3.478	.081
	Rest vs Move	1	.538	.504	Baseline	.820	.379
					DBS	.069	.797
	Interaction	1	3.451	.137			
	Error	4					
	Error (all)	19					

Fig. 8i: 2 x 2 independent model ANOVA

	Main effect	df	F	P	Simple main effect	F	P
MC total power of S(t)	Baseline vs DBS	1	.235	.628	Rest	1.759	.187
					Move	.360	.549
	Rest vs Move	1	3.260	.074	Baseline	.102	.749
					DBS	5.014	.027
	Interaction	1	1.827	.179			
	Error	117					

Fig. 9a: Wilcoxon signed rank test

	Variables	P (2-tailed)
%I<br>30~60 vs 80~100	△V (mV)	.043

Fig. 9e: Friedman tests

	Variables	P (2-tailed)
%I<br>0 vs 30~60 vs 80~100	Burst duration (s)	.007

Fig. 9e: Wilcoxon signed rank test

	Variables	P (2-tailed)
%I<br>0 vs 30~60	Burst duration (s)	.028
%I<br>30~60 vs 80~100	Burst duration (s)	.043
%I<br>0 vs 80~100	Burst duration (s)	.043

Fig. S1a, d: Mann-Whitney U test

	Variables	P (2-tailed)
Control vs.<br>6-OHDA	MC total power	.760
	STN total power	.122

	Variables	P (2-tailed)
Rest vs. Move	STN alpha	.000
	STN delta	.043
	STN beta	.550

Fig. S2a: Mann-Whitney U test

	Variables	P (2-tailed)
Control vs.<br>6-OHDA	Coherence alpha	.138
	Coherence delta	.009
	Coherence beta	.452
